# Multi‐Functional Small Molecule Alleviates Fracture Pain and Promotes Bone Healing

**DOI:** 10.1002/advs.202303567

**Published:** 2023-11-08

**Authors:** Yu‐Ru V. Shih, David Kingsley, Hunter Newman, Jiaul Hoque, Ankita Gupta, B. Duncan X. Lascelles, Shyni Varghese

**Affiliations:** ^1^ Department of Orthopaedic Surgery Duke University School of Medicine Durham NC 27710 USA; ^2^ Department of Mechanical Engineering and Materials Science Duke University Durham NC 27710 USA; ^3^ Translational Research in Pain Program Department of Clinical Sciences College of Veterinary Medicine North Carolina State University Raleigh NC 27607 USA; ^4^ Thurston Arthritis Center University of North Carolina School of Medicine Chapel Hill NC 27599 USA; ^5^ Center for Translational Pain Medicine Department of Anesthesiology Duke University School of Medicine Durham NC 27710 USA; ^6^ Comparative Pain Research and Education Center College of Veterinary Medicine North Carolina State University Raleigh NC 27607 USA; ^7^ Department of Biomedical Engineering Duke University Durham NC 27710 USA

**Keywords:** adenosine, adenosine receptors, biomaterials, drug delivery, fracture healing, fracture pain, phenylboronic acids

## Abstract

Bone injuries such as fractures are one major cause of morbidities worldwide. A considerable number of fractures suffer from delayed healing, and the unresolved acute pain may transition to chronic and maladaptive pain. Current management of pain involves treatment with NSAIDs and opioids with substantial adverse effects. Herein, we tested the hypothesis that the purine molecule, adenosine, can simultaneously alleviate pain and promote healing in a mouse model of tibial fracture by targeting distinctive adenosine receptor subtypes in different cell populations. To achieve this, a biomaterial‐assisted delivery of adenosine is utilized to localize and prolong its therapeutic effect at the injury site. The results demonstrate that local delivery of adenosine inhibited the nociceptive activity of peripheral neurons through activation of adenosine A1 receptor (ADORA1) and mitigated pain as demonstrated by weight bearing and open field movement tests. Concurrently, local delivery of adenosine at the fracture site promoted osteogenic differentiation of mesenchymal stromal cells through adenosine A2B receptor (ADORA2B) resulting in improved bone healing as shown by histological analyses and microCT imaging. This study demonstrates the dual role of adenosine and its material‐assisted local delivery as a feasible therapeutic approach to treat bone trauma and associated pain.

## Graphical abstract

This study reports a translatable approach for local delivery of adenosine and its sustained release in fracture injury. Adenosine demonstrates unique beneficial features for the treatment of fracture injury by simultaneously providing pain relief and promoting bone healing. Specifically, adenosine activates ADORA1 on peripheral neurons to mitigate pain and ADORA2B on osteoprogenitors to promote osteogenesis. This offers an improved approach to manage bone trauma that is unmatched by current therapeutics.

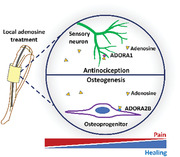

## 1 Introduction

Bone fracture is a common injury associated with sports, accidents, or falls. These injuries and post‐surgical intervention to repair bone are accompanied by acute pain,^[^ ^1^ ^]^ which gradually diminishes as the bone heals.^[^ ^2^ ^]^ The management of bone injuries generally involves stabilization, surgery, rehabilitation, administration of analgesics, such as nonsteroidal anti‐inflammatory drugs (NSAIDs) or opioids, and allowing the injured tissue to heal.^[^ ^1^, ^3^ ^]^ Despite the regenerative capacity of bone, some fractures suffer delayed healing or nonunion, typically requiring repeated surgical interventions, which is a challenging clinical problem and leads to long‐term morbidity and chronic pain.^[^ ^4^ ^]^ The incidence of nonunion varies from 1.9% to 30% depending upon the severity of fracture, comorbidities, and lifestyle habits.^[^ ^4^ ^]^ In addition, the ongoing pain associated with impaired healing often transitions to chronic and maladaptive pain.^[^ ^5^ ^]^

During bone healing, peripheral nerves play an important role in healing outcome. Extensive sensory and sympathetic nerve sprouting occurs after injury/surgery^[^ ^6^ ^]^ and its inhibition negatively affects fracture healing.^[^ ^7^ ^]^ Paradoxically, the growth and activity of peripheral nerves also contribute to pain.^[^ ^5^, ^6^ ^]^ These changes in peripheral nerves could be attributed to an increase in neurotrophins, neuropeptides, and inflammatory molecules that play key roles in fracture healing but also contribute to the induction, sensitization, and maintenance of pain.^[^ ^8^ ^]^ As a result, bone trauma is associated with significant pain requiring the use of analgesics.^[^ ^2^, ^9^ ^]^ While analgesics, such as NSAIDs and opioids, are effective in managing acute pain, their use often leads to unwanted side effects. In addition to interfering with healing, use of NSAIDs and opioids can also result in gastrointestinal irritation and constipation.^[^ ^4^, ^10^ ^]^ Most importantly, post‐operative use of opioids can result in dependence and has been a contributing factor to the current opioid epidemic.^[^ ^4^, ^11^ ^]^ In light of these problems with current analgesics and the lack of osteoanabolic treatments to promote bone tissue regeneration, new approaches that simultaneously manage pain and promote healing would address a significant clinical need. To this end, we examined a new therapeutic solution— localized administration of adenosine— and its efficacy to both mitigate pain and promote tissue regeneration following bone injury or orthopedic surgery.

Adenosine is a naturally occurring nucleoside, and extracellular adenosine regulates tissue function by activating the G‐protein‐coupled adenosine receptors – A1 receptor (ADORA1), A2A receptor (ADORA2A), A2B receptor (ADORA2B), and A3 receptor (ADORA3).^[^ ^12^ ^]^ We and others have demonstrated the key role played by extracellular adenosine in bone health and regeneration,^[^ ^13^ ^]^ and studies have demonstrated impaired fracture healing in *Cd73* (an enzyme that generates extracellular adenosine), *Adora2a*, and *Adora2b* knockout mice.^[^ ^14^ ^]^ We have recently shown that sequestration of extracellular adenosine at the fracture site promoted vascularization and improved fracture repair in a murine tibial fracture model.^[^ ^13c^ ^]^ Similarly, osteoporotic mice treated with an ADORA2B agonist displayed a significant reduction in bone loss.^[^ ^15^ ^]^ In addition to its role in bone repair, adenosine also exhibits analgesic effects and perturbation of adenosine signaling has been shown to play a role in inflammatory and neuropathic pain.^[^ ^16^ ^]^ Activation of ADORA1 and ADORA3 on nociceptors and ADORA3 on spinal microglia have been shown to elicit analgesic effects.^[^ ^17^ ^]^ Targeting pain at the origin of noxious stimuli by local delivery of analgesics could reduce side effects while providing pain relief.^[^ ^18^ ^]^ In this study, we demonstrate that local delivery of adenosine at the fracture site reduces the functional activity of dorsal root ganglion (DRG) neurons through ADORA1, and promotes osteogenic differentiation of mesenchymal stromal cells (MSCs) through ADORA2B. Although adenosine has a rapid clearance and some potential for off‐target, adverse effects when administered systemically, we overcome these drawbacks by employing a biomaterial‐assisted sustained and local delivery of adenosine. In mice with fracture injury, we show that local delivery of adenosine alleviates fracture/post‐surgical pain while promoting bone healing.

## 2 Results

### 2.1 Peripheral Neurons Innervating Fractured Bone Exhibit Enhanced Nociceptive Phenotype

**Figure 1:**
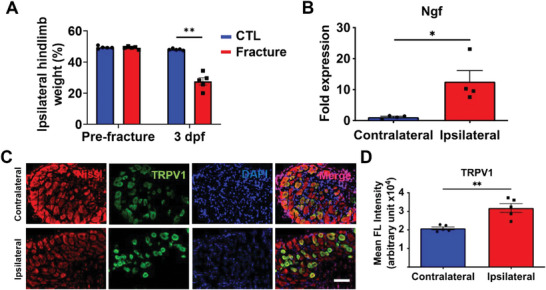
Fracture increases nociception. A) Percent static weight bearing of ipsilateral hindlimb before fracture (pre‐fracture) and 3 days post‐fracture (dpf) of mice with fracture; non‐fractured mice were used as a control (mean ± SEM*, N* = 5 mice per group). B) Relative nerve growth factor (*Ngf*) gene expression of whole bone with marrow compared between contralateral and ipsilateral tibiae of fractured mice (mean ± SEM*, N* = 4 mice per group). C) Immunofluorescence staining of Nissl‐positive neuron cell bodies (red) and TRPV1 (green) in ipsilateral and contralateral L3‐L4 DRG from the fractured mice. 4′,6‐diamidino‐2‐phenylindole (DAPI) stains the nucleus (blue). Scale bar, 200 µm. D) Quantified TRPV1 fluorescence (FL) intensity from the immunofluorescence images (mean ± SEM, *N*>30 DRG neurons per mice, 5 mice per group). Statistical analyses were performed by Mann Whitney U test. *P < 0.05, **P < 0.01.

Injury to bone has been shown to elicit spontaneous and palpation‐induced nocifensive behaviors. These include reduced weight bearing, decreased rearing, and increased mechanical, thermal, and cold allodynia in mice.^[^ ^9^, ^19^ ^]^ Fractures produce an array of inflammatory cytokines, chemokines, neurotrophins, and neurokines that have been shown to contribute to inflammatory pain.^[^ ^8^ ^]^ One such molecule is nerve growth factor (NGF) which is upregulated following fracture and its inhibition has been shown to attenuate fracture pain.^[^ ^7^, ^9^ ^]^ The nociceptive effect of NGF is partially due to its role in promoting the translocation of Transient Receptor Potential Cation Channel Subfamily V Member 1 (TRPV1) to the cell surface membrane and upregulation of TRPV1 expression.^[^ ^20^ ^]^ Studies have found that TRPV1‐expressing neurons in L3 and L4 DRG are pivotal to nociception associated with bone trauma and bone cancer of rodent hindlimbs.^[^ ^21^ ^]^ Since static weight bearing has been used clinically and in animal models to assess fracture pain,^[^ ^22^ ^]^ we used static weight bearing measurements to assess post‐fracture/post‐surgical pain in a mouse (C57BL/6J) model with stabilized unilateral tibial fracture. Ipsilateral hindlimb weight bearing of mice was measured using an incapacitance meter and expressed as percent weight bearing of both hindlimbs. Our results showed that the static weight bearing of ipsilateral (fractured) hindlimb was decreased at 3 days post‐fracture (dpf; **Figure** **1**A). The decreased weight bearing was associated with an upregulation of *Ngf* expression (Figure 1B) and higher levels of TRPV1 expression in ipsilateral Nissl‐positive DRG neurons compared to contralateral DRG neurons (Figure 1C,D). These results suggest that the DRG neurons innervating the fractured limbs exhibit elevated nociception, which is associated with increased *Ngf* and TRPV1 expression.

### 2.2 Adenosine Attenuates NGF‐Induced Sensitization of DRG Neurons through Adenosine A1 Receptor

**Figure 2:**
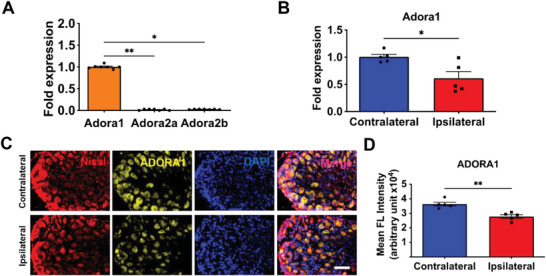
DRG neurons exhibit high expression of adenosine A1 receptor. A) Relative gene expression of adenosine receptors in healthy, non‐fractured L3‐L4 DRG (mean ± SEM, *N* = 7 mice per group. Kruskal‐Wallis with Dunn's post hoc test). B) Relative *Adora1* gene expression of ipsilateral and contralateral L3‐L4 DRG at 3 days post‐fracture (dpf; mean ± SEM*, N* = 5 mice per group. Mann Whitney U test). C) Immunofluorescence images of Nissl‐positive neuron cell bodies (red) and ADORA1 (yellow) in ipsilateral and contralateral L3‐L4 DRG from fractured mice at 3 dpf. DAPI stains nucleus (blue). Scale bar, 200 µm. D) Quantified mean ADORA1 fluorescence (FL) intensity from the immunofluorescence images (mean ± SEM*, N*>20 neurons per mice, 5 mice per group. Mann Whitney U test). *P < 0.05, **P < 0.01.

To determine the relative expression of different adenosine receptor subtypes in DRG neurons, we performed gene expression analyses of whole DRG from healthy, non‐fractured mice. Results showed *Adora1* is expressed at a significantly higher level than *Adora2a*, *Adora2b*, while *Adora3* was undetected (**Figure** **2**A). We next examined the gene expression of DRG from fractured animals, which showed lower expression of *Adora1* in ipsilateral DRG compared to contralateral DRG (Figure 2B). Immunofluorescence staining of ipsilateral and contralateral DRG corroborated the gene expression (Figure 2C,D).

**Figure 3:**
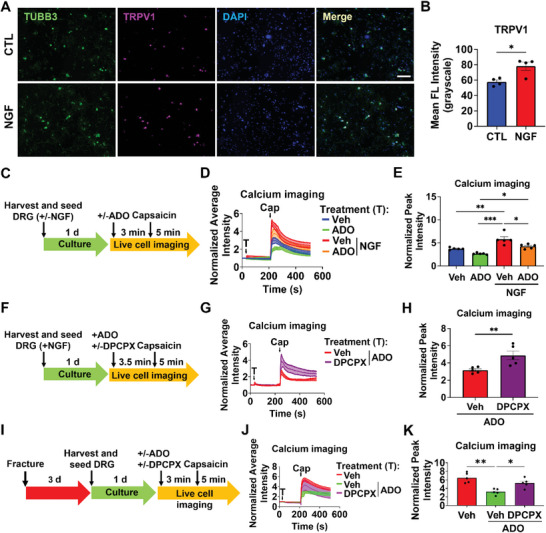
Adenosine attenuates DRG neuron activity through adenosine A1 receptor. A) Immunofluorescence staining of TUBB3 (green) and TRPV1 (violet) in dissociated DRG neurons treated with or without nerve growth factor (NGF; 200 ng mL^−1^). CTL: control. DAPI stains nucleus (blue). Scale bar, 200 µm. B) Quantified TRPV1 fluorescence (FL) intensity from the immunofluorescence images (mean ± SEM, *N*>40 neurons per mice, 4 mice per group. Mann Whitney U test). C) Experimental design of calcium imaging. 1 d: 1 day culture; ADO: adenosine (5 µm). Capsaicin (100 nm). D) Normalized average signal intensity from Fura‐2 calcium imaging. The cells were exposed to various treatments (T) of vehicle (Veh) or adenosine (ADO), and TRPV1 agonist capsaicin (Cap) at the specified time (black arrow). E) Normalized peak signal intensity from Fura‐2 calcium imaging (mean ± SEM, *N*>10 cells per mice, 5 mice per group. Friedman test with Wilcoxon signed‐rank test). F) Experimental design used to examine the role of ADORA1 on the nociceptive function of DRG neurons. 3 d: 3 days. G) Normalized average signal intensity of DRG neurons treated with ADORA1 inhibitor DPCPX compared to the vehicle control (DMSO) from the calcium imaging. Treatments (T) of vehicle (Veh) and DPCPX, and TRPV1 agonist capsaicin (Cap) were added at the specified time (black arrow). H) Normalized peak signal intensity from the calcium imaging (mean ± SEM*, N*>10 cells per mouse, 5 mice per group. Mann Whitney U test). *P<0.05, **P < 0.01, ***P < 0.001. I) Experimental design was used to examine the role of adenosine on the nociceptive function of DRG neurons from the fractured mice. J) Normalized average signal intensity of DRG neurons exposed to DPCPX, treated with or without adenosine, and compared to the vehicle control (DMSO) from the calcium imaging. Treatments (T) of vehicle (Veh), adenosine, DPCPX, and TRPV1 agonist capsaicin (Cap) were added at the specified time (black arrow). K) Normalized peak signal intensity from the calcium imaging (mean ± SEM*, N*>30 cells per mouse, 5 mice per group. Mann Whitney U test). *P<0.05, **P < 0.01.

As *Ngf* expression is known to be elevated in the fractured tibiae, we used NGF as a model molecule for in vitro sensitization of DRG neurons harvested from non‐fractured mice and examined the effect of adenosine to attenuate the increased nociceptive phenotypic and functional changes induced by NGF. Our results demonstrated that one‐day incubation with NGF significantly increased TRPV1 receptor expression in tubulin beta 3 class III (TUBB3)‐positive neurons (**Figure** **3**A,B). Subsequently, calcium imaging was performed on dissociated DRG neurons in the presence or absence of NGF, followed by incubation with or without adenosine as depicted in Figure 3C. The TRPV1 agonist capsaicin is commonly used to assess the functional responsiveness of nociceptive neurons in vitro,^[^ ^23^ ^]^ and DRG neurons in all subsequent imaging experiments were stimulated with capsaicin. Results showed that the exposure of DRG neurons to NGF increased their functional responses to capsaicin, which was attenuated by adenosine (Figure S1, Supporting Information; Figure 3D,E). The adenosine concentration used was identified from an initial screening (Figure S2, Supporting Information). To determine the role of ADORA1 in adenosine‐mediated decrease in functional responses, calcium imaging of DRG neurons exposed to NGF and treated with adenosine in the presence or absence of ADORA1 inhibitor 8‐Cyclopentyl‐1,3‐dipropylxanthine (DPCPX) was performed (Figure 3F). Results showed an increase in the average and peak fluorescence intensity upon treatment with DPCPX compared to the vehicle control lacking DPCPX (Figure 3G,H). We further confirmed the effects of adenosine on neuronal sensitivity by measuring the membrane potential of dissociated DRG neurons in vitro using FluoVolt, a voltage‐sensitive dye (Figure S3, Supporting Information). FluoVolt imaging showed that the NGF exposure increased the membrane potential, but the increase was attenuated in the presence of adenosine. We further examined the effect of adenosine on DRG neurons by using the cells isolated from fractured animals 3 dpf. Calcium imaging was carried out as the DRG neurons were treated with or without adenosine in the presence or absence of ADORA1 inhibitor DPCPX (Figure 3I). Results showed a decrease in the average and peak fluorescence intensity of DRG neurons with adenosine treatment and the decrease was attenuated in the presence of DPCPX (Figure 3J,K).

### 2.3 Local Delivery of Adenosine Improves Weight Bearing

The in vitro results demonstrated the ability of adenosine to inhibit functional responses of DRG neurons including those sensitized by NGF or tibial fracture. We next examined the pain‐relieving effect of adenosine in animals with fracture trauma by local delivery of adenosine following fracture. To aid local delivery, we used poly(ethylene glycol)‐co‐6 aminocaproic acid macroporous hydrogels functionalized with 3‐(acrylamido)phenylboronic acid (3APBA), where the PBA moieties were used to load the adenosine molecules.^[^ ^13c^ ^]^ Details about the fabrication of the macroporous hydrogels are described in Materials and Methods. Successful synthesis of the hydrogel precursors, poly(ethylene glycol) diacrylate (PEGDA) and N‐acryloyl‐6‐aminocaproic acid (A6ACA) from poly(ethylene glycol) (PEG) and 6‐aminocaproic acid (6ACA) respectively, were assessed by FTIR and NMR (Figures S4 and S5, Supporting Information). The fabricated macroporous hydrogel (PEG‐6ACA‐PBA) was characterized by Fourier transform infrared (FTIR) spectroscopy (Figure S6, Supporting Information) and proton nuclear magnetic resonance (^1^HNMR) (Figure S7, Supporting Information) spectroscopy. Adenosine loading was determined by UV/Vis spectrophotometer at a wavelength of 260 nm, which reveals that ≈0.26 ± 0.04 mg adenosine was incorporated per 1 mg of the macroporous hydrogel. The in vitro release kinetics of adenosine from the macroporous hydrogels showed an initial rapid release followed by sustained release (Figure S8, Supporting Information). Following fracture surgery, the animals were implanted with the macroporous hydrogels with or without adenosine.

**Figure 4:**
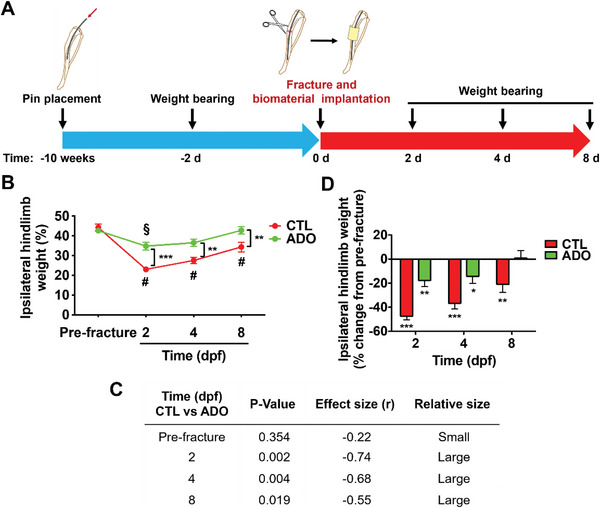
Local delivery of adenosine improves static weight bearing of fractured limbs. A) Schematic illustrating the experimental timeline involving pin placement for stabilization, fracture surgery, biomaterial implantation, and weight bearing experiments. B) Percentage of ipsilateral hindlimb weight bearing in fractured mice treated with control (CTL) or adenosine (ADO)‐loaded macroporous hydrogels up to 8 days post‐fracture (dpf; mean ± SEM, *N* = 9 mice per group. Friedman test with Wilcoxon signed‐rank test was used to compare within the same treatment cohort over time). ^§^ *P* < 0.05, ^#^ *P*<0.01. Mann Whitney U test was used to compare CTL and ADO at the same timepoint. ^**^ *P* < 0.01, ^***^ *P*< 0.001. C) Effect size (r) of treatments on weight bearing corresponding to Figure B. D) Percent change in hindlimb weight bearing up to 8 dpf from pre‐fracture (baseline) (mean ± SEM*, N* = 9 mice per group. Mann Whitney U test was used for each treatment group compared to baseline at each timepoint). *P<0.05, **P < 0.01, ***P < 0.001.

To examine the effect of adenosine on mitigating fracture pain, static weight bearing of mice was measured by incapacitance meter. The experimental timeline is described in **Figure** **4**A. Fracture surgery was performed in the absence of analgesia and macroporous hydrogels measuring ≈4 mm (length) X 2 mm (width) X 1 mm (thick) containing either 1.05 ± 0.1 mg adenosine (ADO) or no adenosine (CTL) were immediately implanted at the fracture site. Ipsilateral hindlimb weight bearing of adenosine and control‐treated mice was expressed as a percent weight bearing of both hindlimbs. Tibial fracture resulted in a significant reduction in ipsilateral weight bearing percentage in the control group at 2, 4, and 8 dpf compared to pre‐fracture (baseline) (Figure 4B). On the other hand, the ipsilateral weight bearing of adenosine‐treated limbs was significantly decreased at 2 dpf and returned back to the baseline by 8 days. The weight bearing of adenosine‐treated limbs was significantly higher compared to control‐treated limbs at all post‐fracture timepoints. Furthermore, the positive impact of adenosine treatment on weight bearing was associated with a large effect size at each timepoint (Figure 4C). The percent change in weight bearing from baseline was also calculated (Figure 4D), which showed significant decrease in weight bearing from the baseline at 2 and 4 dpf for both the treated and untreated groups; however, the degree of decrease in weight bearing was less for the adenosine‐treated group. By 8 dpf, the ipsilateral weight bearing ability of animals treated with adenosine had returned to baseline (i.e., pre‐fracture) while those of the control groups still exhibited significantly reduced weight bearing ability (Figure 4D). To determine whether the locally delivered adenosine resulted in increased adenosine level in the circulation, we measured its concentration in the peripheral blood 3 days after the implantation of adenosine‐loaded macroporous hydrogels at the fracture site. The results showed no significant increase in the adenosine level when compared to control hydrogels without adenosine (Figure S9, Supporting Information).

### 2.4 Local Delivery of Adenosine Improves Open Field Movement

**Figure 5:**
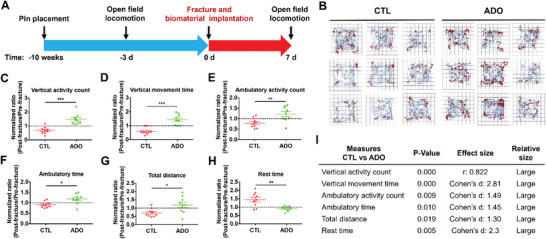
Local delivery of adenosine improves open field activity of fractured animals. A) Experimental timeline of surgery, biomaterial implantation, and open field activity experiments. B) Tracking of vertical activity (red dot) and horizontal activity (blue line) of fractured mice treated with control (CTL) or adenosine (ADO)‐loaded macroporous hydrogels over a 5‐min period at 7 days post‐fracture (dpf). Each grid represents one mouse. C–H) Normalized ratio of total vertical activity count, vertical movement time, ambulatory activity count, ambulatory time, total distance, and rest time of ADO and CTL‐treated mice at 7 dpf. Results are represented as a ratio of post‐fracture divided by pre‐fracture values (mean ± SEM*, N* = 9 mice per group. Mann Whitney U test was used for vertical activity count. Two‐tailed unpaired *t* test was used for other parameters). *P<0.05, **P < 0.01, ***P < 0.001. I) Effect size (r) and Cohen's (d) of treatment on open field activity for each parameter in Figures C–H.

Vertical and ambulatory activity of open field movement are widely used to assess pain and functional outcomes associated with skeletal tissues.^[^ ^24^ ^]^ To assess the changes in ambulatory functions, animals were placed in an enclosed open field and voluntary activities were recorded for a duration of 60 min. The timeline of surgery and open field activity assessment are shown in the experimental design (**Figure** **5**A). Fracture surgery was performed in the absence of analgesia and a macroporous hydrogel with and without adenosine was immediately implanted at the fracture site. The open field test was performed at 7 days post‐fracture and representative plots of activities for each animal (5 min duration between 30th and 35th min) were illustrated by red dots for the vertical activity or rearing, and blue lines for the horizontal activity or ambulation (Figure 5B). The activities for the whole duration were collected as 5‐min intervals and represented as a ratio of post‐fracture to pre‐fracture (Figure S10, Supporting Information). Adenosine‐treated animals had higher levels of activity than the control group for most of the 5‐min intervals including vertical activity count, vertical movement time, ambulatory activity count, ambulatory time, total distance, and lower rest time (Figure S10 and Tables S1–S6, Supporting Information). The sum of activities over the whole duration also showed significantly higher vertical activity counts, vertical movement time, ambulatory activity counts, ambulatory time, total distance, and lower rest time for the mice treated with adenosine compared to the control group (Figure 5C–H). The effect of adenosine treatment was associated with large effect sizes across all of the measured parameters (Figure 5I).

### 2.5 Local Delivery of Adenosine Attenuates Functional Responsiveness of DRG Neurons from Mice with Tibial Fracture

**Figure 6:**
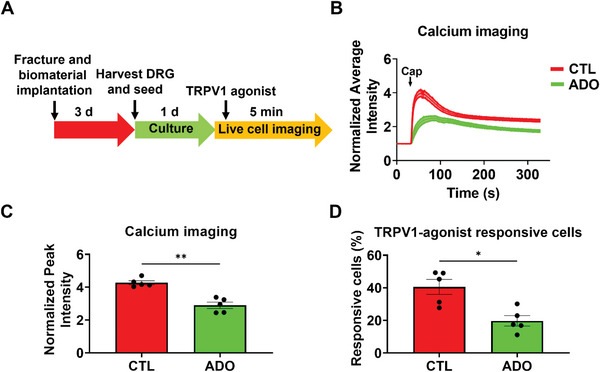
Adenosine treatment attenuates DRG activity of fractured limbs. A) Experimental design for in vitro calcium imaging of dissociated L3‐L4 DRG neurons innervating fractured limbs treated with adenosine (ADO) or control (CTL)‐loaded macroporous hydrogels. B) Normalized average fluorescence (FL) signal intensity from Fura‐2 calcium imaging of DRG neurons innervating fractured hindlimbs treated with ADO or CTL hydrogels. TRPV1 agonist capsaicin (Cap) was added at the specified time (black line) to stimulate cells and signals were normalized to baseline (average of > 30 cells from each mice, 5 mice per group). C) Normalized peak intensity from calcium imaging (mean ± SEM). D) Percentage of DRG neurons from ADO and CTL group that were responsive to capsaicin (mean ± SEM). ADO: adenosine treatment. CTL: Control group without adenosine treatment. Statistical analyses were performed using a Mann Whitney U test. *P < 0.05, **P < 0.01.

To examine the in vivo effect of adenosine on the functional responsiveness of DRG neurons from mice with fractured tibiae, L3‐L4 DRG were harvested 3 days post‐fracture from animals that were implanted with hydrogels with or without adenosine, and cultured for 1 d prior to calcium imaging (**Figure** **6**A). Results showed DRG neurons associated with adenosine‐treated limbs had lower average and peak fluorescence intensity compared to DRG neurons from untreated control animals (Figure 6B,C). The percentage of DRG neurons that were responsive to capsaicin from adenosine‐treated animals were also significantly lower compared to controls (Figure 6D). This finding mirrors the results from weight bearing and open field activity experiments and suggests that the adenosine‐mediated decrease in neuronal responsiveness is responsible for reduced pain and improved limb function in adenosine‐treated animals.

### 2.6 Local Delivery of Adenosine Promotes Fracture Healing

**Figure 7:**
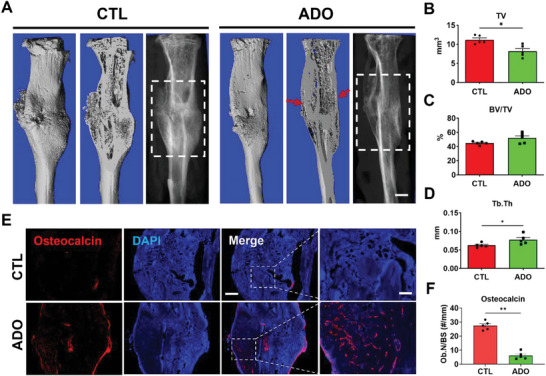
Local delivery of adenosine promotes fracture repair. A) 3D reconstructed images (intact and cut) and radiographs from microCT imaging of fractured tibiae treated with adenosine (ADO) or control (CTL) at 21 days post‐fracture (dpf). Red arrow indicates connected cortical bone. Scale bar, 2 mm. Quantification of B) total volume (TV), C) percent bone volume (BV/TV), and D) trabecular thickness (Tb.Th) of regenerating calluses from microCT images. White dashed box highlights the callus region. E) Osteocalcin staining and F) quantification for number of positive cells on bone surface (Ob.N/BS) in fractured tibiae treated with adenosine (ADO) or control (CTL)‐loaded hydrogels at 21 dpf. (mean ± SEM*, N* = 5 mice per group. Mann Whitney U test). *P < 0.05. Scale bar, 2 mm. Magnified image scale bar, 500 µm.

Microcomputed tomography (microCT) images were used to assess fracture healing at 21 days post‐fracture. Results showed local delivery of adenosine accelerated fracture healing as demonstrated by 3D reconstructed microCT images (intact and cut views) and corresponding radiographs of the fractured tibiae of the adenosine‐treated groups demonstrate improved bridging of cortical bone with less trabecular bone (red arrows), and smaller calluses (highlighted white dash box), akin to our prior study (**Figure** **7**A).^[^ ^13c^ ^]^ Quantification of microCT images indicated lower total volume, higher bone volume ratio, and increased trabecular thickness in cohorts treated with adenosine compared to corresponding control group (Figure 7B–D). Similar to microCT analyses, safranin O histological images showed more cortical bone across the fracture site (red arrows) and smaller callus size in adenosine‐treated groups compared to controls at 21 days post‐fracture (Figure S11, Supporting Information). Smaller total volume and callus size indicate better bone healing.^[^ ^25^ ^]^ Osteocalcin staining of the calluses showed higher number of osteocalcin‐positive osteoblasts on bone surfaces in the adenosine‐treated cohorts indicating more bone‐formation (Figure 7E,F). On the other hand, tartrate‐resistant acid phosphatase (TRAP) staining for osteoclast activity was lower in the adenosine‐treated cohorts compared to controls (Figure S12, Supporting Information).

### 2.7 Adenosine A2B Receptor is Highly Expressed in Osteoprogenitors of Regenerating Bone

**Figure 8:**
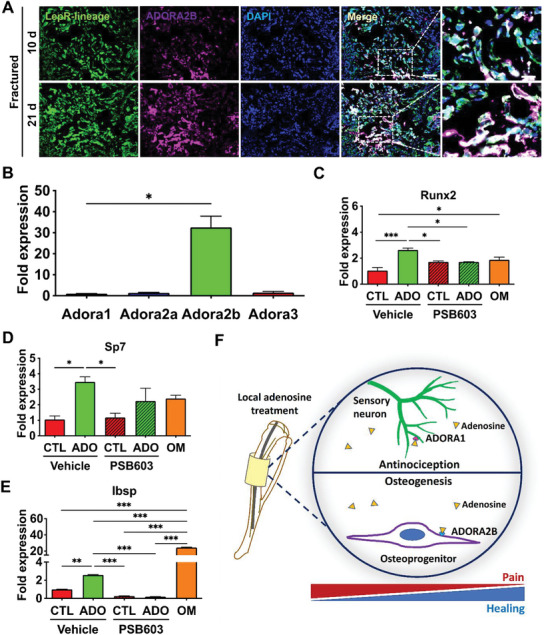
Osteoprogenitor cells exhibit high expression of adenosine A2B receptor. A) Immunofluorescence staining of Td‐Tomato‐labeled LepR lineage cells (green) and ADORA2B (violet) in regenerating bone of fractured mice at 10 and 21 days post‐fracture (dpf). DAPI stains nucleus (blue). Scale bar, 100 µm. Magnified image scale bar, 25 µm (*N* = 3 mice). B) Relative gene expression of adenosine receptors in bone marrow mesenchymal stromal cells (BM‐MSCs; mean ± SEM*, N* = 3 mice pooled per group. Kruskal‐Wallis test with Dunn's post hoc test). C–E) Gene expression of *Runx2*, *Sp7*, and *Ibsp* in BM‐MSCs, respectively. Cells were treated with adenosine (ADO, 30 µg mL^−1^) or control (CTL) in the presence of vehicle (DMSO) or ADORA2B inhibitor PSB 603 for 14 d. OM: osteogenic media. (mean ± SEM*, N* = 3 pooled mice per group. Friedman test with Wilcoxon signed‐rank test). *P < 0.05, **P < 0.01, ***P < 0.001. F) Proposed action of adenosine on multiple cell populations and adenosine receptor subtypes resulting in reduced fracture pain and improved healing.

Given the key role played by ADORA2B in osteogenic differentiation of stem cells^[^ ^14^, ^26^ ^]^ and fracture healing,^[^ ^14c^ ^]^ we examined the expression of ADORA2B in progenitor cells in the fracture callus. The leptin receptor (LepR) has been demonstrated to be a marker of bone marrow progenitor cells.^[^ ^27^ ^]^ These cells proliferate and serve as a cell source for bone tissue formation following injury.^[^ ^27a^ ^]^ Using *Lepr‐cre;tdTomato* conditional reporter mice to trace LepR(+) lineage cells, we examined the expression of ADORA2B in LepR lineage progenitors after fracture by immunofluorescent staining for ADORA2B. Our results showed abundant co‐localization of ADORA2B expression in LepR lineages in the fracture calluses at 10 and 21 days post‐fracture (**Figure** **8**A). Furthermore, gene expression analyses of adenosine receptors in bone marrow‐derived MSCs demonstrated significantly higher levels of *Adora2b* expression compared to other adenosine receptor subtypes (Figure 8B). We also examined the role of ADORA2B activation on osteogenic differentiation of MSCs. Results showed that adenosine upregulated gene expression of osteoblast transcription factors, *Runx2* and *Sp7*, and osteoblast marker, *Ibsp*, in the absence of osteogenic induction medium (Figure 8C–E). This adenosine mediated upregulation of osteogenic markers was attenuated in the presence of ADORA2B‐specific inhibitor PSB 603 (Figure 8C–E). We also examined the expression levels of different adenosine receptors in the bone marrow and bone tissues of contralateral and ipsilateral tibiae. Results showed expression of all four adenosine receptors in contralateral and ipsilateral bone and bone marrow tissues and expression levels ranked from highest to lowest were: *Adora1*>*Adora3*>*Adora2b*>*Adora2a* (Figure S11A, Supporting Information). The expression level of *Adora2b* in the bone marrow slightly increased during regeneration (Figure S11B, Supporting Information).

## 3 Discussion

Fractures and associated pain (including post‐surgical pain) are common clinical problems. Currently, NSAIDs and opioid analgesics are used to treat fracture pain. While these approaches are effective in managing pain, they can be associated with various side effects including interference with healing. Considering the limitations associated with NSAIDs and opiates, development of novel therapies to attenuate fracture pain without interfering with healing is highly desirable. In this study, we examined the efficacy of local delivery of adenosine to attenuate fracture pain while promoting fracture healing with improved limb function. Our findings show that adenosine can simultaneously elicit osteoanabolic and pain‐mitigating functions. Specifically, adenosine activates ADORA1 on sensory neurons and inhibits neuronal excitation to mitigate pain, while activating ADORA2B on osteoprogenitors to promote osteogenesis and fracture healing (Figure 8F). Although pharmacological activation of individual adenosine receptor subtypes using synthetic agonists is a viable strategy to either attenuate fracture pain or promote healing, we used adenosine due to its broader beneficial effects and ability to target multiple adenosine receptor subtypes and different cell populations.

We observed increased TRPV1 expression in DRG neurons after injury, and higher expression and functional activity of dissociated DRG neurons cultured in presence of NGF, a neurokine that is elevated in the fracture microenvironment.^[^ ^7^, ^9^, ^28^ ^]^ TRPV1 has been shown to play a significant role in inflammatory pain^[^ ^29^ ^]^ that sensitizes C and Aδ fiber bone afferent neurons.^[^ ^21a^ ^]^ Its elevated expression after bone injury correlates with elevated levels of several inflammatory molecules.^[^ ^8^, ^21^ ^]^ The increased DRG neuronal function was attenuated in the presence of adenosine and pharmacological inhibition studies demonstrated the central role played by ADORA1. Studies have shown *Adora1* knockout mice experience higher sensitivity to heat and cold, and ADORA1 activation has an analgesic effect in inflammatory and neuropathic pain.^[^ ^30^ ^]^ This effect is due to ADORA1‐mediated inhibition of cyclic AMP and PKA,^[^ ^31^ ^]^ and induction of neuronal hyperpolarization by activating K^+^ channels while inhibiting Ca^2+^channels.^[^ ^32^ ^]^ Recent transcriptomic studies of human DRG neurons also suggest a role played by *ADORA1* in functional activity.^[^ ^33^ ^]^ The established anti‐nociceptive effect of ADORA1 activation along with findings that *Adora1* gene expression is expressed at significantly higher levels than other adenosine receptor subtypes in the mouse DRG suggest that it plays a dominant role in inhibiting fracture pain. This result is consistent with findings in the literature which showed that *Adora1* RNA expression is significantly more abundant in mouse DRG while the other adenosine receptor subtypes have minimal or undetectable expression.^[^ ^34^ ^]^ Our experiments revealed a decrease in *Adora1* expression in DRG following bone injury. A similar observation of decreased ADORA1 expression was reported in spinal cords after spinal cord injury.^[^ ^35^ ^]^ We speculate that the reduced expression of ADORA1 could partially be responsible for pain following injury, bestowing a protective mechanism to guard the fractured tissue. Together, the significantly higher expression of ADORA1 compared to other adenosine subtypes in DRG neurons and in vitro findings that adenosine inhibits NGF‐ and TRPV1‐induced nociception through ADORA1 supports its role in adenosine‐induced pain relief post‐fracture/post‐surgery.

Similarly, studies have shown inflammatory molecules also contribute to pain, and inhibiting such molecules in itself prevents inflammatory nociception.^[^ ^36^ ^]^ Since adenosine is a pro‐resolving mediator that modulates inflammatory response of immune cells through ADORA2A, ADORA2B, and ADORA3,^[^ ^37^ ^]^ its indirect effect on anti‐nociception through regulation of inflammatory mediators cannot be ruled out. Besides inflammatory pain, adenosine has been shown to be effective against neuropathic pain^[^ ^38^ ^]^ and can act on both the CNS and PNS,^[^ ^17^, ^39^ ^]^ suggesting its broad applications in pain relief. Despite the potential of adenosine to mitigate pain,^[^ ^40^ ^]^ it has not been adopted as a therapeutic due to the ubiquitous expression of adenosine receptors throughout the body, a narrow therapeutic window, and its rapid clearance in circulation.^[^ ^41^ ^]^ Its short half‐life and broad effects in the body^[^ ^12a^ ^]^ suggest that adenosine was designed by nature to act locally to exert a rapid regulation of the immediate environment. With that in mind, we have leveraged a biomaterial‐assisted local and sustained delivery of adenosine to regulate adenosine signaling over a prolonged period of time.^[^ ^13c^ ^]^

Our in vivo studies demonstrate that biomaterial‐assisted local delivery of adenosine attenuates fracture/surgical pain in the absence of other analgesics. Specifically, weight bearing of the fractured limb treated with adenosine was significantly higher with large effect sizes suggesting less pain. Of note, some pain from pin placement appeared to be present as ipsilateral hindlimb weight bearing percentage was 45% and not at 50% as expected, though prior studies have suggested pain returns to baseline after 4 weeks.^[^ ^9b^ ^]^ Similarly, open field activity of animals treated with adenosine was also significantly improved and demonstrated a large effect size indicating adenosine not only reduced pain but also improved limb function. Pain diminishes after bone heals, yet the pain relief by adenosine treatment is due to its analgesic effect and not due to its effect on bone healing as the nociceptive responses were assessed at early timepoints. Together the open field activity and weight bearing measurements suggest sustained pain reduction and improved limb function in treated animals.

In addition to anti‐nociception, the ubiquitous expression of multiple adenosine receptor subtypes present in various cell types within the bone tissue offers other potential benefits such as the ability to promote bone regeneration. As expected, osteoblasts were more abundant on bone surfaces in the regenerating callus of adenosine‐treated cohorts as shown by osteocalcin staining. However, TRAP expression was reduced in the adenosine‐treated cohorts compared to controls. This could be due to the advanced progression of healing in the adenosine‐treated calluses compared to control calluses as osteoclast activity is expected to be predominantly high during the middle phase of healing corresponding to resorption of the cartilaginous soft callus and beginning of the remodeling of hard bone tissue.^[^ ^42^ ^]^ Previous studies have shown that fracture healing and osteogenic differentiation of MSCs were impaired in *Adora2b* knockout mice.^[^ ^14c^ ^]^ However, the expression of ADORA2B in osteoprogenitors during fracture repair in vivo remained undetermined. In this study, we demonstrate co‐localization of ADORA2B in LepR osteoprogenitors in vivo, further corroborating the key role of ADORA2B in osteogenesis of MSCs to promote healing. This is consistent with our previous studies which showed adenosine promotes osteogenesis (and diminishes adipogenesis) of human BM‐MSCs through ADORA2B.^[^ ^26a,c^ ^]^ While we have focused on ADORA2B signaling, the contribution of ADORA2A in MSCs towards bone regeneration cannot be ruled out in this study.^[^ ^14b^ ^]^ As immune cells, including alternatively activated (M2‐like) macrophages, have been shown to be important for bone repair,^[^ ^43^ ^]^ and A2 receptors (ADORA2A and ADORA2B) promote their polarization,^[^ ^44^ ^]^ it is likely that adenosine also induces a pro‐regenerative immune environment by regulating immune cells through A2 receptors.^[^ ^43^ ^]^

Although the anti‐nociceptive effect of adenosine/adenosine receptor agonists has been shown in multiple animal models, a recent study demonstrated that adenosine deaminase‐deficient mice with sustained systemic elevation of extracellular adenosine exhibited chronic pain.^[^ ^34^ ^]^ Accumulation of high levels of adenosine activated myeloid cells through ADORA2B and contributed to chronic pain through a proinflammatory milieu including IL‐6 signaling. The increased pain observed could be due to the significantly high levels of adenosine in the circulation associated with the global adenosine deaminase‐deficient animal model. In this study, we have used a biomaterials‐assisted local delivery of adenosine to restrict the signaling at the site of injury. Sustained, local delivery of adenosine can circumvent side effects associated with bolus and/or systemic delivery such as cardiovascular side effects and vasodilatory effects.^[^ ^12^, ^41^ ^]^ Recent studies have used nanocarriers and/or tissue‐targeting as an approach to increase accumulation of adenosine or adenosine receptor agonist at the bone tissue following their systemic administration.^[^ ^45^ ^]^ While such drug delivery approaches could improve accumulation of adenosine to the bone tissue, the nanocarriers can be up taken by other organs such as liver and spleen. Thus, locally implanted biomaterial such as the one used in this study or an injectable in situ curing biomaterial^[^ ^45b^ ^]^ to deliver adenosine to the organ or tissue would be ideal to improve its therapeutic efficacy. Taken together, these studies suggest both the therapeutic potential of local delivery of adenosine in managing pain and the potential to be translated into human patients, but the latter can only be concluded after a clinical trial.

Alleviation of pain by non‐addictive analgesics that do not interfere with healing will have a significant clinical impact as it could reduce the use of NSAIDs and opioids and thus prevent their adverse effects on bone healing and potentially combat the opioid crisis. While results from the described study show that local modulation of adenosine signaling and targeting adenosine subtypes in different cell populations in bone tissue could hold promise as an effective therapy for bone repair, additional studies need to be further explored. First, as the activity of other adenosine receptor subtypes in inflammatory cells has been shown to be involved in nociception,^[^ ^16^, ^37^ ^]^ the indirect role of adenosine receptors in immune cells to reduce pain could not be elucidated in this study. Second, the current study clearly demonstrates the beneficial effect of adenosine on acute pain, however, its effectiveness for chronic pain conditions such as nonunions remains to be examined.

## 4 Conclusion

Biomaterial‐assisted local delivery of adenosine in fracture injury prolongs its therapeutic effect and demonstrates unique beneficial features to simultaneously promote bone healing while providing pain relief. Specifically, adenosine activates ADORA1 on peripheral neurons to mitigate nociception and ADORA2B on osteoprogenitors to promote osteogenesis. These unique features of adenosine offer an improved approach to address bone trauma that is unmatched by current therapeutics.

## 5 Experimental Section

### Study Design

The aim of the study was to examine the effectiveness of local delivery of adenosine in tibial bone injury to concurrently attenuate fracture/post‐surgical pain while promoting healing. All animal work was performed in compliance with the National Institutes of Health (NIH) and institutional guidelines, and the fracture surgery and biomaterial implantation protocols were approved by the Institutional Animal Care and Use Committee at Duke University (Protocol Registry Number A151‐20‐07). C57BL/6J mice were used to study fracture nociception and the effect of local delivery of adenosine to mitigate pain and promote bone regeneration. *Lepr‐cre; tdTomato* reporter mice were used for tracing of LepR(+) lineage cells after fracture. To determine the effect of adenosine on pain and healing, fracture surgeries were performed and treated immediately with PEGDA‐6ACA‐PBA macroporous hydrogels loaded with adenosine as treatment group or without adenosine as control group.^[^ ^13c^ ^]^ Behavioral tests for pain and limb function were assessed by static weight bearing and open field locomotion tests. A priori power analysis was carried out based on the pilot and previous studies to estimate the sample size requiring a statistical power (1‐β) of 80% at the significance level of *α* = 5%. Ten animals per group were used to study the effect of adenosine on fracture pain, and 5 animals per group to examine the effect of fracture on pain, and adenosine treatment on fracture healing. The exclusion criteria for experiments were animals that died due to the surgical procedures or shift of the intramedullary pin identified after experimental endpoint post‐mortem. Measurement of adenosine in the circulation was performed by using an adenosine assay.^[^ ^13^, ^15^ ^]^ The in vitro functional activity of DRG neurons was assessed by calcium and membrane potential imaging and the expression of proteins was assessed by histology or immunofluorescence imaging, and gene expression by RT‐qPCR. Fracture healing was analyzed by microCT and histology. The study design and timeline of animal procedures (pin placement, fracture surgery, therapeutic intervention with and without adenosine, and behavioral tests) are illustrated in Figure 4A (weight bearing) and Figure 5A (open field locomotion). The sample number, and statistical methods for each experiment are provided in the figure legends.

### Fracture Surgery and Therapeutic Intervention

In all experiments, 12‐ to 16‐week‐old C57BL/6J male mice (Jackson Laboratory, Bar Harbor, ME) and *Lepr‐cre; tdTomato* reporter mice were used. The average mouse weight was 25 g and mice were housed in groups of four to five, and kept on a 12‐h light–dark cycle with lights off at 1800 h. All animals had ad libitum access to chow (PMI LabDiet 5001) and water. Tibial fracture surgeries were performed as previously described.^[^ ^13c^ ^]^ Animals were anesthetized by isoflurane and injected with buprenorphine SR‐LAB (1 mg kg^−1^; ZooPharm, Laramie, WY). Each mouse was then placed in a supine position with the right tibia disinfected. After skin incision and reflection, proximal to the right knee, a 0.7‐mm pin was inserted from the tibial plateau through the medullary cavity to stabilize the tibia, and a cut was made at the tibial midshaft to induce a transverse fracture. Bupivacaine (0.5%; Hospira, Lake Forest, IL) at ≈1 mg kg^−1^ body weight was applied at the surgical site following wound closure using 2 reflex clips (5 mm). For animals treated with control and adenosine‐loaded macroporous hydrogels, macroporous hydrogels measuring approximately 5 mm (length) X 3 mm (width) X 1 mm (thick) containing either 1.05 ± 0.1 mg adenosine (ADO) or no adenosine (CTL) were implanted at the fracture site immediately. For behavioral tests, fracture surgery was delayed until 10 weeks after the pin placement to avoid interference from pain originating from sites other than the fracture site, as the pin insertion alone has been shown to induce pain.^[^ ^9b^ ^]^

### Statistical Analyses

Statistical analyses were carried out using GraphPad Prism 9 or SPSS v.20. Mann‐Whitney U test was used to compare between two groups. Statistical analysis comparing multiple groups was performed by Kruskal‐Wallis with Dunn's post hoc test or Friedman test with Wilcoxon signed‐rank test, as appropriate. For all comparisons, two‐tailed tests were used and *P* <0.05 was considered statistically significant. Cohen's *d* or *r* was used to calculate standardized effect size using SPSS v.20, as appropriate. Power analysis was performed using G*Power.

## Conflict of Interest

S.V., Y.V.S., and J.H. are inventors on patent publication US 2020/036012, and S.V. and Y.V.S. are inventors on Provisional Application 63/297216 held/submitted by Duke University for this study. The authors declare that they have no other competing interests.

## Supporting information

Supporting Information

## Data Availability

The data that support the findings of this study are available from the corresponding author upon reasonable request.
