# Supplementary material for: Multi‐Functional Small Molecule Alleviates Fracture Pain and Promotes Bone Healing
Source: Adv Sci (Weinh). 2023 Nov 8;10(36):2303567. doi: 10.1002/advs.202303567 (PMC10754086; doi:10.1002/advs.202303567)
Supplement: Supplementary file 1 — Supporting Information [file ADVS-10-2303567-s001.pdf]

ADVANCED SCIENCE
Open Access

# Supporting Information

for *Adv. Sci.*, DOI 10.1002/advs.202303567

Multi-Functional Small Molecule Alleviates Fracture Pain and Promotes Bone Healing

*Yu-Ru V. Shih*, David Kingsley, Hunter Newman, Jiaul Hoque, Ankita Gupta, B. Duncan X. Lascelles and Shyni Varghese**

# Supporting Information

## Multi-functional small molecule alleviates fracture pain and promotes bone healing

*Yu-Ru V. Shih*, David Kingsley, Hunter Newman, Jiaul Hoque, Ankita Gupta, B. Duncan X. Lascelles, Shyni Varghese**

**Content**

Materials and Methods
Figure S1 to S13
Table S1 to S6

## Materials and Methods

### Synthesis and characterization of macroporous hydrogels

*Synthesis of polyethylene glycol diacrylate (PEGDA):* Poly(ethylene glycol) was acrylated as described elsewhere.[34] In brief, polyethylene glycol (PEG; 10 g, ~3 mmol; MilliporeSigma, Burlington, MA, Cat# P4338) was dissolved in dry dichloromethane (DCM, 100 mL) at room temperature under argon gas. Triethylamine (627.3 µL, 4.5 mmol; MilliporeSigma, Cat# 471283) was added to the solution. The reaction mixture was then placed on an ice bath. Acryloyl chloride (364 µL, 4.5 mmol; MilliporeSigma, Cat# A24109), dissolved in dry DCM (15 mL), was added dropwise to the mixture and the reaction was continued for about 12 h at room temperature. The reaction mixture was then passed through Celite 545 (MilliporeSigma, Cat# 1026931000) and concentrated using a rotary evaporator. The product was precipitated in excess chilled diethyl ether and filtered using Whatman filter paper. The resultant PEGDA was dried overnight under vacuum and purified by using Sephadex G-25 column (GE Healthcare, Chicago, IL) followed by lyophilization. The PEGDA product was characterized by a combination of FTIR and $^{1}$HNMR spectroscopy. The FTIR spectra showed peaks at 1725 cm$^{-1}$ corresponding to the ester C=O stretching frequency thus confirming the introduction of acrylate groups *via* ester bond formation (Figure S4A). The diacrylation in PEGDA was further confirmed from $^{1}$HNMR, which showed the presence of peaks at 5.81, 6.31 and 6.42 ppm corresponding to vinyl protons of the acrylate groups (Figure S4B).

*Synthesis of N-acryloyl-6-aminocaproic acid (A6ACA):* A6ACA was synthesized as described earlier [46]. 6-Aminocaproic acid (6ACA; 13.1 g, 0.1 M; MilliporeSigma, Cat# A7824) and sodium hydroxide (4.4 g, 0.11 M) was dissolved in water (80 mL). The solution was placed over an ice bath and acryloyl chloride (~10 g, 0.11 M) dissolved in dry tetrahydrofuran (THF; 15 mL) was added to the 6ACA solution dropwise. The pH of the reaction mixture was maintained at ~7.8 during the addition of acryloyl chloride by using NaOH (2.5 M) solution. After the reaction, the pH of the mixture was gradually decreased to ~3.0 by adding hydrochloric acid (5 M). The product was extracted using ethyl acetate, concentrated over anhydrous sodium sulfate, and precipitated in chilled n-hexane. The product, A6ACA, was filtered through Whatman filter paper and dried overnight under vacuum at 45°C. The product was characterized by FTIR and $^1$HNMR spectroscopy. The FTIR spectrum showed peaks at 1655 $cm^{-1}$ and 1543 $cm^{-1}$ corresponding to the amide C=O and N−H stretching frequencies, respectively (Figure S5A). Formation of A6ACA was further evident from $^1$HNMR spectrum which showed peaks at 5.74, 6.18 and 6.26 ppm corresponding to vinyl protons of acrylamide group (Figure S5B).

*Macroporous hydrogel fabrication:* The PEGDA-6ACA macroporous hydrogels containing 3-acrylamido phenylboronic acid (3-APBA; MilliporeSigma, Cat# 771465) (PEGDA-6ACA-PBA) were fabricated using a poly(methyl methacrylate) (PMMA) bead leaching method.[13c] PEGDA (10% w/v), 3-APBA (1 M), A6ACA (0.5 M) and Irgacure 2959 (MilliporeSigma, Cat# 410896; 0.5% w/v) were dissolved in 20:80 water:ethanol mixture. A mixture of 20 µL was added into a cylindrical polypropylene mold (~5 mm in diameter) packed with ~20 mg of PMMA microbeads (150-180 µm; Cospheric, Santa Barbera, CA). The mixture was photopolymerized by UV light irradiation for about 10 min. The resulting hydrogel network embedded with the PMMA beads was incubated in acetone for 3 days to remove the beads with frequent changes of solvent. The macroporous hydrogels were washed and rehydrated with deionized water. FTIR spectrum of the freeze-dried hydrogel showed a peak at 1725 $cm^{-1}$ corresponding to the ester C=O stretching indicating the presence of PEGDA (Figure S6). Presence of phenylboronic acid (PBA) moieties was confirmed *via* $^1$HNMR spectroscopy. For NMR measurements, freshly prepared macroporous hydrogels were thoroughly washed with DI water to remove the unreacted precursors and freeze-dried. The freeze-dried samples were minced and fully suspended in $D_2O$ by adding NaOH solution (5 M) in $D_2O$. The $^1$HNMR spectrum, recorded using a 500 MHz

Varian spectrometer, showed peaks at 7.07-7.27 ppm corresponding to aromatic protons of PBA (Figure S7). The PBA content in the hydrogel was quantified by using UV/Vis spectrophotometer. Towards this, the macroporous hydrogels were dissociated in NaOH solution (5 M) in water. The PBA content in the solution was determined by using a UV/Vis spectrophotometer at an absorption wavelength of 255 nm. A standard calibration curve of absorbance *vs.* concentration was generated using free 3-APBA solutions of known concentrations (21.25 to 170 µg $mL^{-1}$) and used to calculate the PBA content. The estimated value suggests that the PBA constituted over 45% of the dry weight of hydrogels which further indicated that almost 93±3% of 3-APBA was incorporated into the hydrogel network. Macroporous hydrogels without 3-APBA (PEGDA-6ACA) were prepared similarly and used as controls for animal studies. For sterilization, the hydrogels were soaked in 70% ethanol for 6 h and washed extensively in phosphate buffered saline (PBS) for 3-4 days.

*Adenosine loading:* To load adenosine (MilliporeSigma, Cat# A4036) into the macroporous hydrogel (PEGDA-6ACA-PBA), hydrogel discs were incubated in adenosine solution (6 mg/mL) in PBS (20 mL) for about 6 h at 37 °C and washed thoroughly to remove any unbound adenosine. To measure the amount of adenosine loaded, the discs were soaked in acetate buffer (0.1 M, pH 3.5) for about 2 h to release the adenosine into the buffer. The adenosine content in the buffer was determined by using a UV/Vis spectrophotometer at wavelength of 260 nm. A standard calibration curve of absorbance *vs.* concentration was generated using adenosine solutions of known concentrations (3.9-125 µg $mL^{-1}$) and used to calculate the adenosine concentration.

*Adenosine release:* To determine the release profile of adenosine, adenosine loaded macroporous hydrogels were incubated in α-MEM (ThermoFisher Scientific, Waltham, MA, Cat# 12561056) containing fetal bovine serum (FBS, 10% v/v) at 37 °C. At predetermined time intervals, 20 vol% of the medium was removed and supplemented with fresh medium. The concentration of adenosine in the released medium was determined *via* UV/Vis absorption spectroscopy using a standard calibration curve as described above.

## Behavioral tests

In the cohorts used for behavior measurements with macroporous hydrogel implantation, 1 out of 10 animals from the control group died following surgery and 1 out of 10 animals from the treatment group was euthanized due to shift of the inserted pin following fracture.

*Weight bearing*

Static weight bearing was measured using an incapacitance meter (IITC Life Science, Woodland Hills, CA). Prior to measurements, mice were trained on the incapacitance meter for 5 days (10 min per day). Data were collected if the animal stood in an upright position and facing front, without noticeable weight shift, lifting or offloading a limb, or turning the head. Each animal was tested for 5 to 6 trials and the weight of both hind limbs was recorded. Weight bearing of the fractured limb (i.e., ipsilateral, right limb) was expressed as a percentage of the total weight borne by the hindlimbs calculated by: (weight of ipsilateral hindlimb/total weight of both hindlimbs)*100.

*Open Field Activity*

Open field activity was performed at Duke Mouse Behavioral and Neuroendocrine Core similar to ref. [47]. Mice were acclimated to the room for a day prior to testing. Mice were placed individually into the VersaMax open field activity monitoring system with clear acrylic test chambers (40 X 40 X 30 cm) and a grid of infrared photobeams containing photocells and sensors to collect and analyze vertical, horizontal, and stereotypic activity (AccuScan Instruments, Columbus, OH). Locomotion was monitored over 60 min at 5 min intervals. The vertical activity count, vertical movement time (s), ambulatory activity count, ambulatory time (s), total distance traveled (cm), and rest time (s) were determined by the software. Results were expressed as a ratio of post-fracture divided by pre-fracture (baseline) values.

## MicroCT

Tibiae were collected, fixed in PFA (4%) at 4 °C for 1 day, and rinsed with PBS. The fixed samples were placed in 50-mL centrifuge tubes with styrofoam spacers and loaded into a microcomputed tomography (microCT) scanner (vivaCT 80, Scanco Medical, Wayne, PA). The samples were scanned at 55 keV at a pixel resolution of 10.4 μm. Reconstruction of the images was performed using microCT Evaluation Program V6.6 (Scanco Medical), followed by

generation of radiographs and 3D models using microCT Ray V4.0 (Scanco Medical). Total volume (TV), bone volume ratio (BV/TV), and trabecular thickness (Tb.Th) was quantified by microCT Ray V4.0 using the phantom as a reference based on 100 contiguous slices.

**Cell isolation and *in vitro* culture**

*DRG neurons:* DRG were isolated from mice using a modified version of the previously described protocol.[48] Briefly, mice were euthanized and doused in ethanol (70%) to prevent fur contamination. Mice were positioned prone, and an incision was made along the spine from the neck to the base of the tail. Under a dissecting microscope, the surrounding soft tissue of the spinal column was removed from the dorsal side by Friedman-Pearson Rongeurs (Fine Science Tools, Foster City, CA; Cat# 16021-14) from the mid thoracic region to the lumbosacral joint. The vertebral bone surrounding the DRG was gently crushed and removed with rongeurs exposing the spinal cord. The DRG were carefully extracted near the foramen between the vertebral levels. L3 and L4 DRG were collected for analyses. Dissected DRG were placed directly into ice-cold DMEM-F12 medium (ThermoFisher, Cat# 11320033) for in vitro culture, Trizol (ThermoFisher, Cat# 15596018) for PCR analyses, or PFA (4%) for histology. For cell culture experiments, collected DRG were digested in digestion solution comprised of collagenase type II (Worthington, Lakewood, NJ, Cat# LS004176) and dispase (1.5 mg $mL^{-1}$ each) in DMEM/F12. DRG were agitated in the digestion solution on an orbital shaker at 60 rpm and 37°C for 20 min and repeated thrice with fresh digestion solution. Next, the digestion solution was replaced with trypsin-EDTA (0.025%) in DMEM/F12 and incubated for an additional 15 min to disrupt the remaining cell-to-cell adhesions. The solution was then replaced with DMEM/F12 and FBS (1:3) to neutralize trypsin. DRG were triturated to create a cell suspension and gently layered onto bovine serum albumin (BSA, 15%) solution without mixing and centrifuged for 6 min at 280 rcf with minimal acceleration and no deceleration to separate non-neuronal cells and debris.[49] The supernatant was carefully aspirated, and the sensory neurons were resuspended in DRG culture medium. To culture DRG neurons, culture media composed of Neurobasal-A medium (ThermoFisher, Cat# 10888022), B27 supplement (2%; ThermoFisher, Cat# 17504044), glutamax (1%; ThermoFisher, Cat# 35050061), and penicillin/streptomycin (10000 U/mL, 1% v/v; ThermoFisher, Cat# 15140122) was used. Cells were treated with or without NGF (200 ng $mL^{-1}$) for 24 h prior to immunofluorescence imaging, or calcium and

FluoVolt imaging. Cells were cultured in a custom-made well containing silicone walls and coverslip bottom. Briefly, coverslips (#1 thickness) were cleaned by dipping in NaOH (0.5 M) for 30 min followed by subsequent rinsing in $dH_2O$ and ethanol (100%), and air-dried. Cured polydimethylsiloxane (PDMS, Sylgard 184, Ellsworth Adhesives, Germantown, WI; Cat# 3097358-1004) with an 8-mm hole (generated using an 8-mm biopsy punch) was fabricated and bonded to the cleaned coverslips to create the cell culture well. The coverslips were coated with poly-lysine by treating overnight with poly-d-lysine solution (0.1 mg $mL^{-1}$), rinsed with sterile water and air-dried. These custom-fabricated cell culture dishes were stored for up to 7 days prior to use. The dishes were further treated with laminin (20 µg $mL^{-1}$) for at least 4 hours prior to cell seeding. Laminin solution was aspirated out, and incubated with culture medium for 1 hour prior to culturing DRG neurons.

*MSC:* MSCs were isolated as previously described with some modifications.[15] Briefly, the femurs, tibiae, and vertebrae of mice were harvested, crushed with pestle and mortar in harvest buffer (1% v/v FBS in PBS) to release bone marrow (BM) tissue, filtered through a 40-µm cell strainer, and centrifuged at 200 rcf. Cells were seeded in a 24-well plate at a cell density of 1 million cells per $cm^2$ and cultured in growth media (GM) of α-MEM, containing FBS (10% v/v; ThermoFisher, Cat# 16000044), penicillin/streptomycin (10000 U/mL, 1% v/v) in a humidified incubator (37°C, 5% $CO_2$). The medium was replaced after 3 days and cultured for an additional 6 days. For passaging, cells were incubated in trypsin-EDTA (0.25%) for 2 min at 37°C, detached with a cell scraper, neutralized by using GM, centrifuged, and sub-cultured at a density of 8000 cells per $cm^2$. All experiments were performed between 1-2 passages. Osteogenic medium (OM) was prepared by supplementing GM with β-glycerophosphate (10 mM; MilliporeSigma, Cat# G9422), ascorbic acid-2-phosphate (50 µM; MilliporeSigma, Cat# A8960), and dexamethasone (100 nM; MilliporeSigma, Cat# D4902).

**Calcium imaging**

Intracellular cytosolic $Ca^{2+}$ was evaluated by Fura-2 loaded DRG neurons.[50] Fura-2 (ThermoFisher, Cat# F1221) reagents (2 mM in 100% DMSO) were mixed with Pluronic F-127 at 1:1 volume ratio, and then mixed with Tyrode's solution (140 mM NaCl, 5 mM KCl, 2 mM $CaCl_2$, 2 mM $MgCl_2$, 10 mM HEPES, 10 mM glucose, pH 7.4) at 1:500 volume ratio.[50] DRG samples were washed twice in Tyrode's solution before being replaced with Fura-2 solution and

incubated at room temperature for 40 min. Samples were washed thrice with Tyrode's solution and incubated for an additional 20 min prior to mounting for imaging. Cells were mounted on the translation stage of an Olympus IX81 inverted microscope (Olympus America) and depending on the experimental group, were treated with either adenosine (5 μM), or adenosine (5 μM) along with ADORA1 inhibitor 8-Cyclopentyl-1,3-dipropylxanthine (DPCPX; 100 nM) for 5 min prior to stimulating with capsaicin (100 nM). For the experiments involving DPCPX, since DPCPX was dissolved in DMSO a corresponding control culture exposed to the vehicle DMSO was used.

Fura-2 dual excitation and emission were accomplished using 340- and 380-nm excitation filters and a 510 nm emission filter and imaged with an Olympus UPlan FLN 20X 1.3 NA water immersion objective. Light was supplied by a Lambda XL (Sutter Instrument Company, Novato, CA) using variable aperture. Digital images (150-ms exposure) were recorded with a Hamamatsu EM CCD camera (Hamamatsu Photonics, Hamamatsu City, Japan) at 1-s intervals. Imaging was performed by first establishing a baseline intensity ratio (340/380 nm) for the region of interest (ROI) prior to capsaicin (TRPV1 agonist) treatment. The normalized Fura-2 intensity profiles were plotted as ratiometric intensity divided by the baseline intensity. Peak intensity measurements are reported as maximum normalized intensity during stimulation. The total number of DRG neurons in a given ROI was determined by adding potassium chloride (KCl; 80 mM) at the end of the experiment and counting the total number of activated DRG neurons, i.e., cells with a ratiometric change.

**Membrane potential imaging**

DRG neuron potential changes were evaluated by using FluoVolt, a voltage sensitive indicator dye (ThermoFisher, Cat# F10488). Cells were stained with FluoVolt according to manufacturer instructions; briefly, the staining solution was prepared by adding 10X component B (10 μL) and component A (1 μL) in a 1.5-mL tube, followed by Tyrode's solution (1 mL). DRG culture medium was removed and washed prior to adding the loading solution and incubated at room temperature for 30 min. FluoVolt staining solution was then removed and washed thrice with Tyrode's solution. Imaging was performed using an Olympus IX81 inverted microscope (Olympus America), with FITC excitation filter. Digital images (250-ms exposure) were recorded with a Hamamatsu EM CCD camera (Hamamatsu Photonics) at 1-s intervals. Imaging was performed prior to capsaicin stimulation to determine the baseline intensity for the DRG

neurons. Fluorescence intensity profiles were reported as the intensity at a given time point divided by the baseline intensity. Peak intensity measurements are reported as the maximum normalized intensity during stimulation.

**RT-qPCR**

Cells, DRG, or bone/marrow tissues were analyzed for gene expression by quantitative real-time polymerase chain reaction (RT-qPCR). Nucleic acids were extracted with TRIzol, phase-separated with chloroform, and precipitated in isopropanol. One microgram of RNA was reverse transcribed using iScript cDNA Synthesis Kit (Bio-Rad, Hercules, CA, Cat# 1708891) according to the manufacturer's instructions.[45a] Quantitative PCR was performed with iTaq Universal SYBR green reagent (Bio-Rad, Cat# 1725124) with denaturation at 95°C for 30 s for one cycle, and amplification (denaturation + annealing/extension) at 95°C for 5 s and 60°C for 30 s for 40 cycles using a polymerase chain reaction (PCR) cycler (Bio-Rad, CFX96 Touch). The primer sequences used are: *Ngf* (forward, GGGAG CGCAT CGAGT TTTG; reverse, TACGC TATGC ACCTC ACTGC), *Adora1* (forward, CCCCA TCGTCTA TGCCTTCC; reverse, CATCG GAAGT GGTCG TTCCA), *Adora2a* (forward, GCCAG AGCAA GAGGC AGGTA T; reverse, TCCCA AAGGC TTTCT CACGG), *Adora2b* (forward, ATCTT TAGCC TCTTG GCGGT G; reverse, GACCC AGAGG ACAGC AATGA T), *Adora3* (forward, GCTGTA GACCGA TACCTG CG; reverse, GGAAAC TAGCCA GCAAAG GC), *Runx2* (forward, TGGCC GGGAA TGATG AGAAC; reverse, TGAAA CTCTT GCCTC GTCCG), *Sp7* (forward, TGCCT GACTC CTTGG GACC; reverse, TAGTG AGCTT CTTCC TCAAG CA), *Ibsp* (forward, TCCAC ACTTT CCACA CTCTC G; reverse, CTTTC TGCAT CTCCA GCCTT C), *18S ribosomal RNA* (forward, ACCAG AGCGA AAGCA TTTGC CA; reverse, ATCGC CAGTC GGCAT CGTTT AT), *Gapdh* (forward, GCACA GTCAA GGCCG AGAAT; reverse, GCCTT CTCCA TGGTG GTGAA).[14] The expression level of each target gene was normalized to the housekeeping gene and to their respective controls and presented as fold change, $2^{-\Delta\Delta Ct}$ values. *Ngf* expression was normalized to *Gapdh*, and other gene expressions were normalized to *18S rRNA*.

**Immunofluorescence staining of DRG**

*In vitro* cultured DRG neurons and frozen DRG tissue sections were stained and imaged. *In vitro cultured DRG neurons:* cells were fixed in PFA (4%) for 15 min, permeabilized in Triton X-100 (0.1%) in PBS for 10 min, followed by blocking for 1 h in BSA (3%) in PBS at room temperature. Cells were co-stained with TUBB3 (Novus Biologicals, Cat# NB100-1612, 1:500 dilution in 3% BSA) and TRPV1 (Novus Biologicals, Cat# NBP1-71774, 1:300 dilution in 3% BSA) antibodies at 4°C overnight. Cells were washed with PBS at room temperature for 10 min thrice and incubated with secondary antibodies donkey anti-chicken AlexaFluor 488 (Jackson ImmunoResearch, Cat# 703-545-155, 1:300 dilution) and donkey anti-rabbit AlexaFluor 647 (Jackson ImmunoResearch, Cat# 711-605-152, 1:300) for 1 h at room temperature. *Frozen DRG tissue sections:* L3-L4 DRG were dissected, fixed with 4% PFA at 4°C for 2 h, incubated in 30% sucrose overnight, embedded in OCT, and 5-μm thick sections were generated by using a cryostat (Leica, CM1850). Sections were heated for antigen retrieval in citrate buffer (MilliporeSigma, Cat# C9999) for 20 min, permeabilized in 0.1% Triton X-100 in PBS for 10 min, followed by blocking for 1 hour in blocking solution comprised of BSA (3%), glycine (0.26 M), normal donkey serum (5%) in Tris buffered saline (TBS) at room temperature. Sections were stained with TRPV1 (Novus Biologicals, Cat# NBP1-71774, 1:300 dilution in blocking solution) antibody, or ADORA1 (Proteintech, Cat# 55026-1-AP, 1:100 dilution in blocking solution) antibodies at 4°C overnight. Sections were washed with Tris buffered saline (TBS) with Tween-20 (0.1%; TBS-T) at room temperature for 10 min thrice, and then incubated with secondary antibody donkey anti-rabbit AlexaFluor 647 (Jackson ImmunoResearch, Cat# 711-605-152, 1:300 dilution in blocking solution) for 1 h at room temperature. Nissl (ThermoFisher, Cat# N21482, 1:100 dilution in PBS) was used to stain DRG neurons at room temperature for 20 min. Finally, all samples were washed with TBS-T, covered with mounting solution (ThermoFisher, Cat# P36971), sealed, and imaged using TRITC, Cy5, GFP, and DAPI filters on a Keyence BZ-X710 microscope. Fluorescence intensity was quantified by ImageJ software and presented as arbitrary units.

**Histological staining of tibiae**

Tibiae were processed for subsequent immunofluorescence staining of Td-tomato and ADORA2B, osteocalcin, and histochemical staining of safranin O and TRAP. Briefly, tibiae

were fixed with PFA (4%) at 4°C for 1 day and decalcified using ethylenediaminetetracetic acid (EDTA; 14%, pH 7.3) for 2 weeks at 4°C with constant shaking. The samples were gradually dehydrated using increasing concentrations of ethanol and incubated in Citrisolv (Decon Laboratories, King of Prussia, PA) until equilibrium was reached. Following dehydration, samples were immersed in a mixture of Citrisolv (50% v/v) and paraffin (50% w/w; General Data Healthcare, Cincinnati, OH) for 30 min at 70 °C. The samples were embedded in paraffin and 7-μm thick sections were generated using a rotary microtome (Leica Microsystems, Buffalo Grove, IL, RM2255). *Immunofluorescence staining of Td-tomato and ADORA2B:* sections were heated for antigen retrieval in citrate buffer (MilliporeSigma, Cat# C9999) for 20 min, permeabilized in Triton X-100 (0.1%) in PBS for 10 min, followed by blocking for 1 h in a blocking solution comprised of BSA (3%), glycine (0.26 M), normal donkey serum (5%) in TBS at room temperature. Sections were co-stained with Td-tomato (MyBiosource, Cat# MBS448092, 1:100 dilution in blocking solution) and ADORA2B (MyBiosource, Cat# MBS8207549, 1:100 dilution in blocking solution) antibodies at 4°C overnight. Sections were washed with TBS-T at room temperature for 10 min thrice, and then incubated with secondary antibodies donkey anti-goat FITC (Jackson ImmunoResearch, Cat# 705-096-147) and donkey anti-rabbit AlexaFluor 647 (Jackson ImmunoResearch, Cat# 711-605-152, 1:300) for 1 h at room temperature. *Immunofluorescence staining of osteocalcin:* rehydrated tissue sections were incubated with 10 μg/mL proteinase K, immersed in a blocking buffer (TBS, normal donkey serum [10%], Tween-20 [0.5%]) for 1 h, and incubated with osteocalcin primary antibody (MilliporeSigma, Cat# AB10911) diluted 1:400 in antibody diluent buffer (TBS, normal donkey serum [5%], Tween-20 [0.1%]) overnight at 4°C. After rinsing thoroughly, the sections were incubated with donkey anti-rabbit AlexaFluor 647 secondary antibody (Jackson ImmunoResearch, Cat# 711-605-152) diluted 1:300 in antibody diluent at room temperature for 1 h. All immunofluorescence stainings were washed with TBS-T (TBS, 0.1% Tween-20), covered with mounting solution (ThermoFisher, Cat# P36971), sealed, and imaged using Cy5, GFP, or DAPI filters on a Keyence BZ-X710 microscope. The number of osteoblasts was quantified by counting the number of osteocalcin-positive cells over the length of bone surface (Ob.N/BS) using ImageJ. *Safranin O staining:* To visualize the callus remodeling and bone tissue formation, tissue sections were stained with safranin-O (1%; Millipore Sigma, Cat# S8884) for 1 h and counter-stained with Fast Green (0.02%; Millipore Sigma, Cat# F7258) for 1 min. The sections were then

dehydrated in an ethanol gradient solvent and mounted with Cytoseal (ThermoScientific, Cat# 23-244256). *TRAP staining:* TRAP staining were performed as previously described [45a]. Sections rinsed with distilled water were incubated in acetate buffer (0.2 M) containing sodium L-tartrate dibasic dihydrate (50 mM; MilliporeSigma, Cat# 228729-100G) at pH 5.0 for 20 min at RT followed by incubation with naphthol ASMX phosphate disodium salt (0.5 mg/mL; MilliporeSigma, Cat# N5000-1G) and Fast Red TR Salt 1,5-naphthalenedisulfonate (1.1 mg/mL; MilliporeSigma, Cat# F6760-5G) for 60 min at 37 °C in dark. TRAP staining was quantified using ImageJ as percent area of positive stain over total area (Oc.S/BS [%]). All histochemical stainings were imaged using a Keyence BZ-X710 microscope.

**Measurement of adenosine in plasma**

Peripheral blood was collected and immediately incubated in ice-cold stop solution at a 1:2 ratio (blood to stop solution) to inhibit degradation of adenosine.[51] The stop solution is comprised of dipyridamole (0.2 mM; Tocris, Minneapolis, MN), erythro-9(2-hydroxy-3-nonyl)-adenine (5 μM; EHNA; Tocris), Adenosine 5'-(α,β-methylene) diphosphate sodium salt (62 μM; APCP), EDTA (5 mM), and heparin (25 IU $mL^{-1}$; Tocris) in PBS. The blood was centrifuged at 2000 rcf for 10 min at 4°C to separate cells from plasma. Adenosine assay kit (Cell Biolabs, Cat# MET-5090) was used to measure adenosine levels according to manufacturer's protocol. Briefly, a reaction mixture containing the fluorometric Probe, HRP, adenosine deaminase, purine nucleoside phosphorylase, xanthine oxidase, assay buffer and a control mixture comprised of fluorometric Probe, HRP, purine nucleoside phosphorylase, xanthine oxidase, assay buffer were made and mixed with plasma (50 μL) for 15 min. at room temp. The relative fluorescence unit (RFU) was measured using a microplate reader with excitation and emission at 570 nm and 590 nm, respectively. Adenosine concentration was determined using a standard curve of known concentrations and subtracted for the background by using the values in control mix.

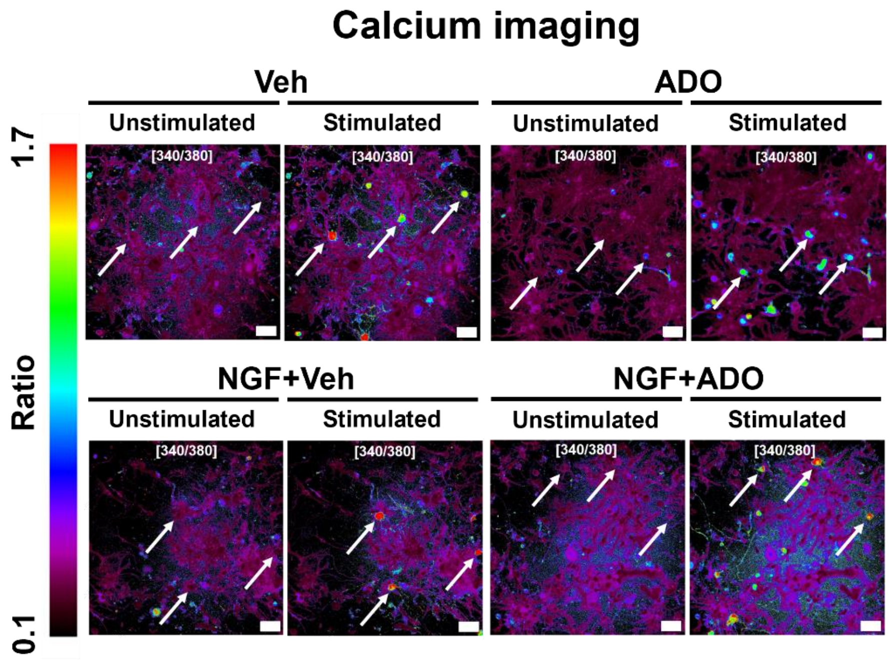


**Figure S1. Representative images from calcium imaging using Fura-2 dye.** TRPV1 agonist capsaicin was used to stimulate the cells and signals were normalized to the baseline. Arrows indicate cells with changing fluorescence after stimulation with capsaicin.

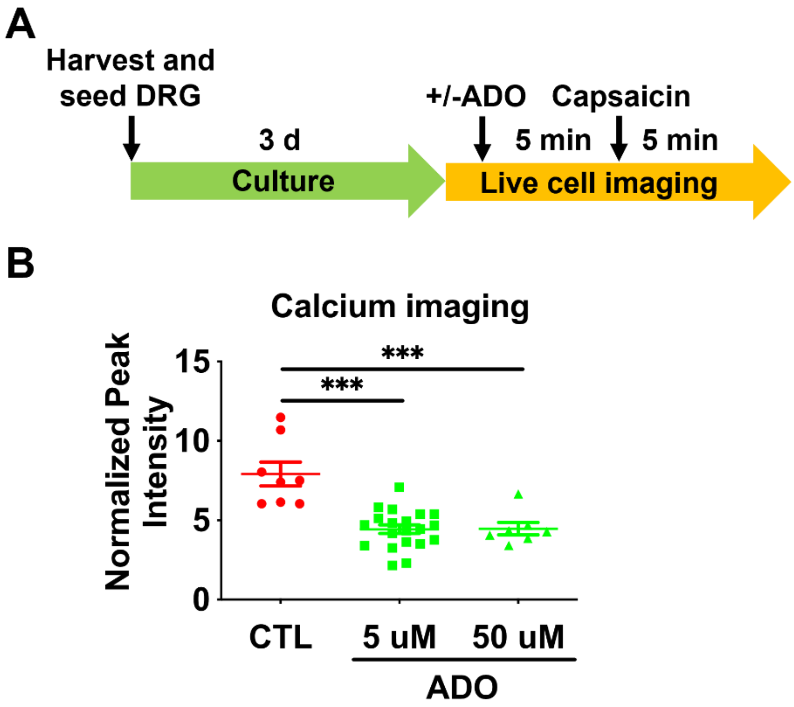


**Figure S2. Extracellular adenosine decreases functional activity of mouse DRG neurons.** (**A**) Experimental design of calcium imaging. (**B**) Normalized peak intensity of dissociated DRG neurons *in vitro* treated with two different concentrations of adenosine (ADO) and stimulated by TRPV1 agonist capsaicin (mean ± SEM, $N$>7 cells per group. Two-tailed unpaired $t$ test). ***$P$ < 0.001.

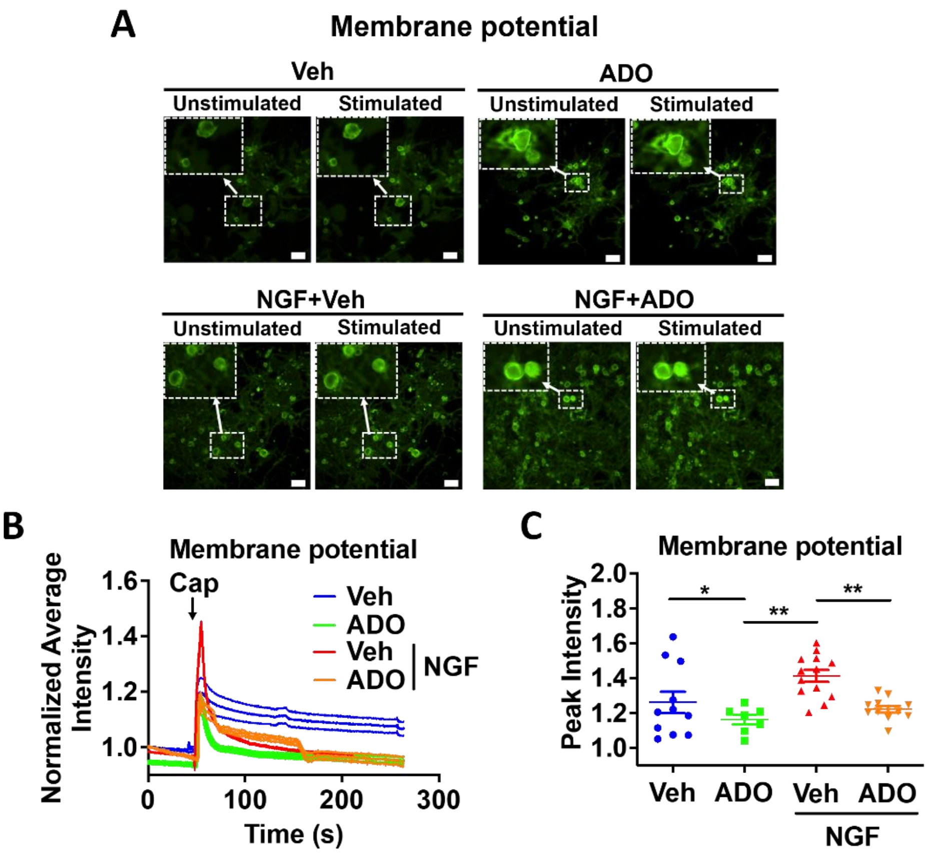


**Figure S3. Adenosine attenuates NGF-induced increase in membrane potential of DRG neurons.** (**A**) Relative fluorescence intensity of membrane potential imaging of dissociated DRG neurons after 1 d of NGF treatment followed by adenosine treatment. Magnified views indicate cells with changing fluorescence intensity after stimulation with TRPV1 agonist capsaicin (Cap), which was added at the specified time (black arrow). (**B**) Normalized average signal intensity from membrane potential imaging. (**C**) Normalized peak intensity from membrane potential imaging (mean ± SEM, *N*=7-13 cells per group. Two-way ANOVA with Tukey post hoc test). *$P$<0.05, **$P$ < 0.01.

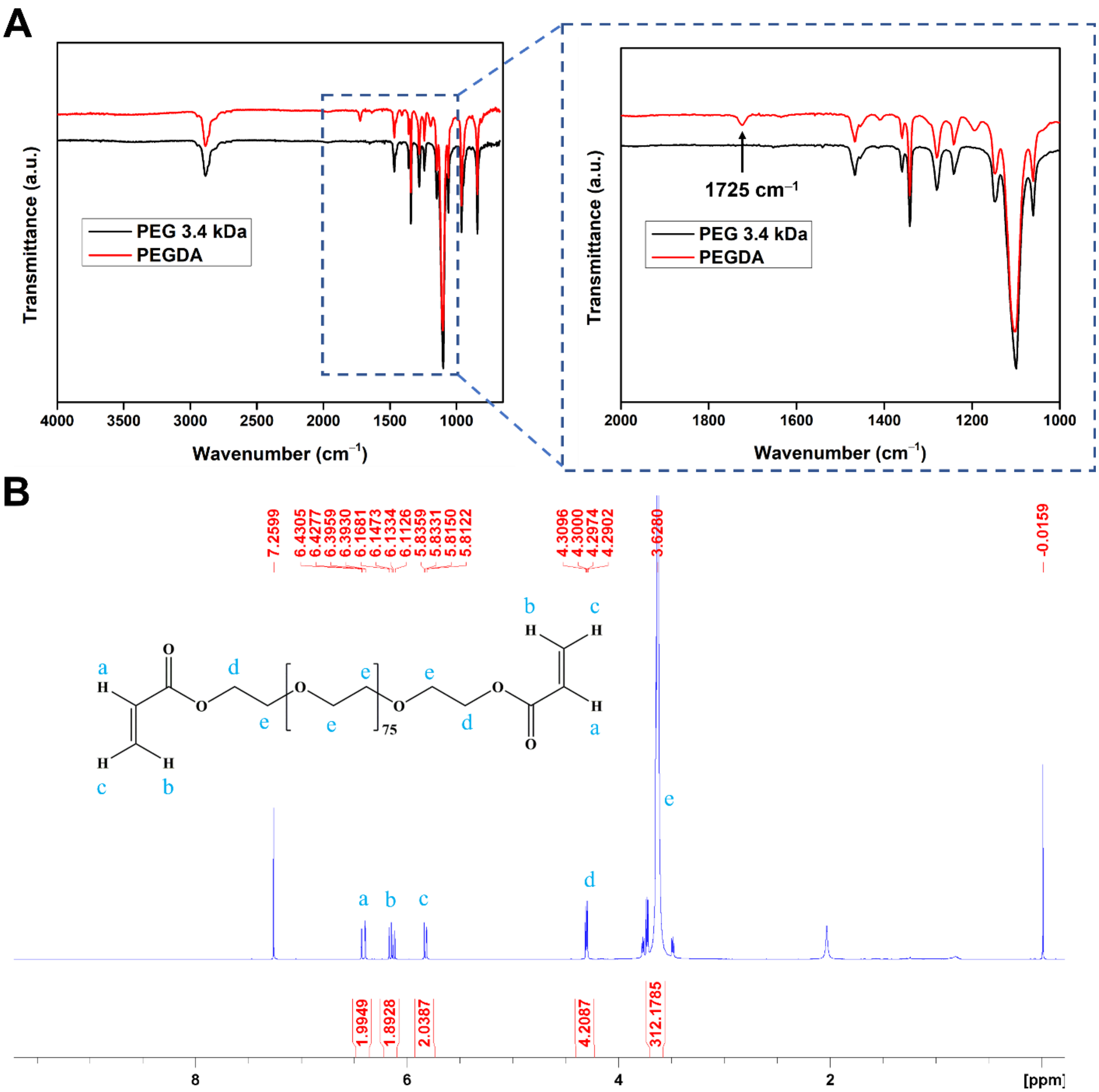


**Figure S4. Characterization of poly(ethylene glycol diacrylate) PEGDA.** (A) FTIR and (B) $^{1}$HNMR spectrum of PEGDA. FTIR spectra were recorded using ZnSe crystal in attenuated total reflectance (ATR) mode. Arrow at 1725 cm$^{-1}$ indicates the stretching frequency of the ester C=O bond in PEGDA. For $^{1}$HNMR spectrum, PEGDA was dissolved in $CDCl_3$.

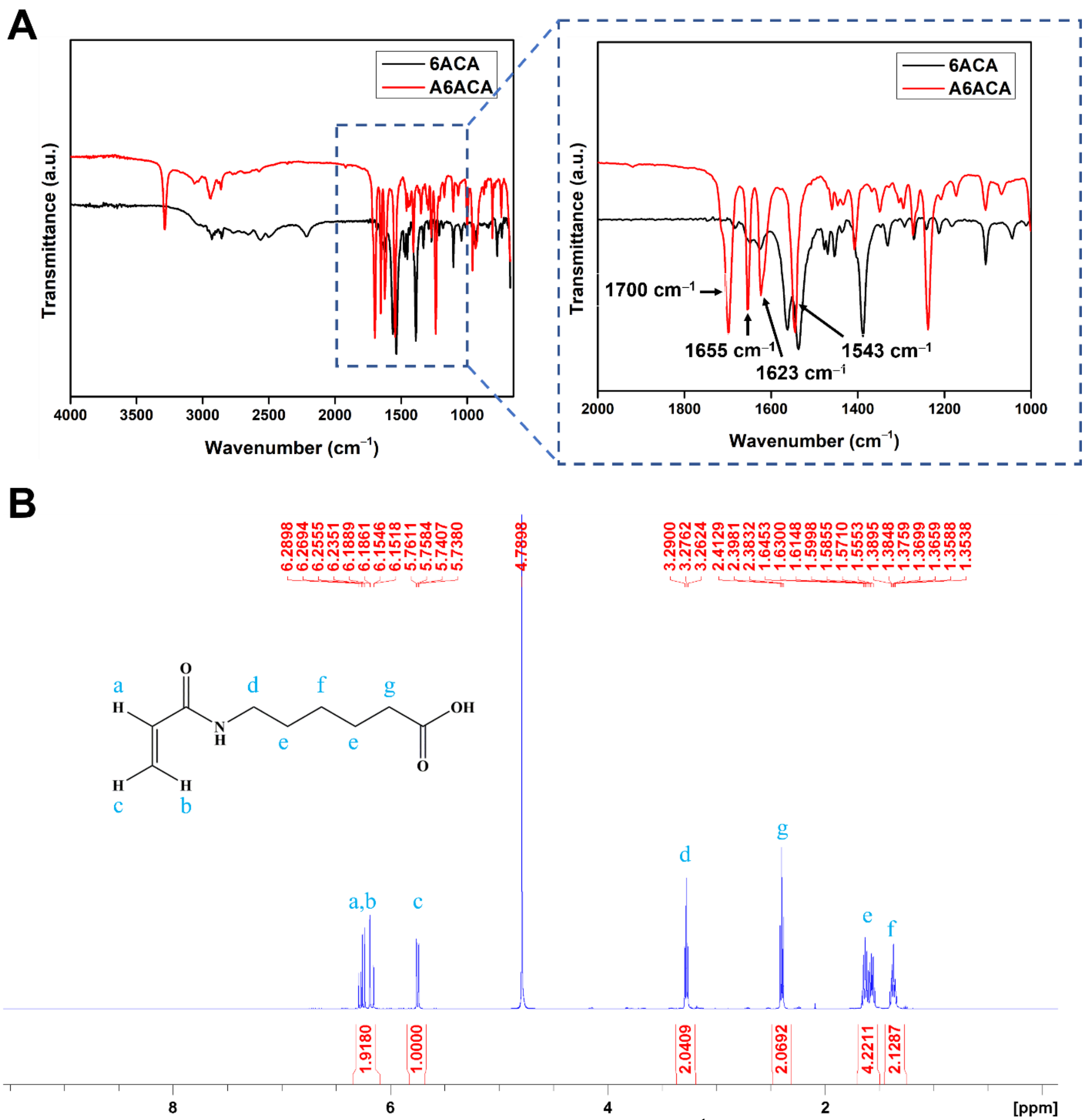


**Figure S5. Characterization of A6ACA.** (A) FTIR and (B) $^{1}$HNMR spectrum of Acryloyl-6-aminocaproic acid (A6ACA). FTIR spectra were recorded using ZnSe crystal in attenuated total reflectance (ATR) mode. Arrows at 1655 cm$^{-1}$ and 1543 cm$^{-1}$ correspond to the amide C=O and N–H stretching frequencies, respectively. For $^{1}$HNMR spectrum of A6ACA was dissolved in $D_2O$.

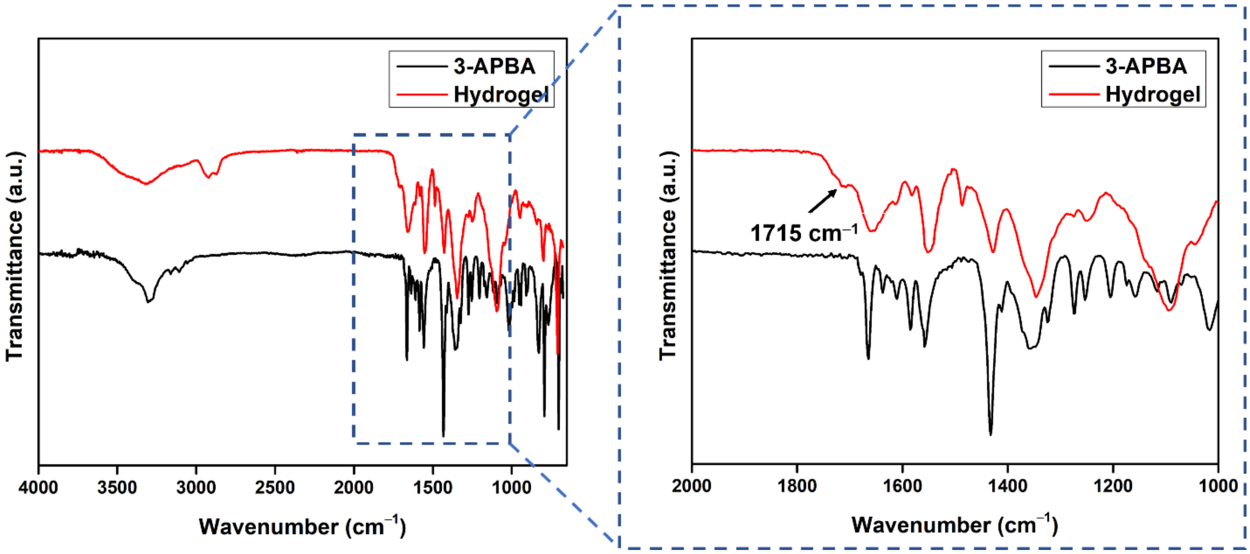


**Figure S6. Characterization of PEGDA-6ACA-PBA macroporous hydrogel.** FTIR spectra of the lyophilized macroporous hydrogel recorded using ZnSe crystal in attenuated total reflectance (ATR) mode. Arrow at 1715 $cm^{-1}$ indicates the stretching frequency of the ester C=O bond of PEGDA in PEGDA-6ACA-PBA hydrogel.

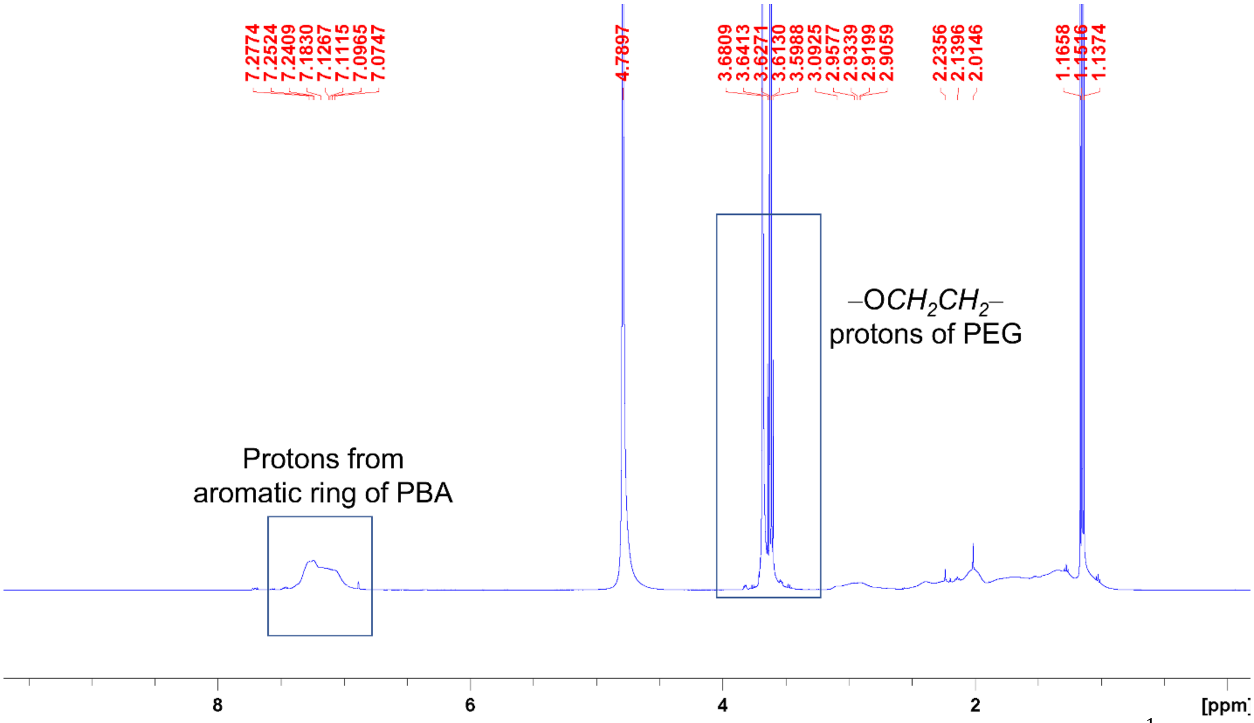


**Figure S7. Characterization of PEGDA-6ACA-PBA macroporous hydrogel.** $^{1}$HNMR spectrum of the macroporous hydrogel in $D_2O$ at 25 °C.

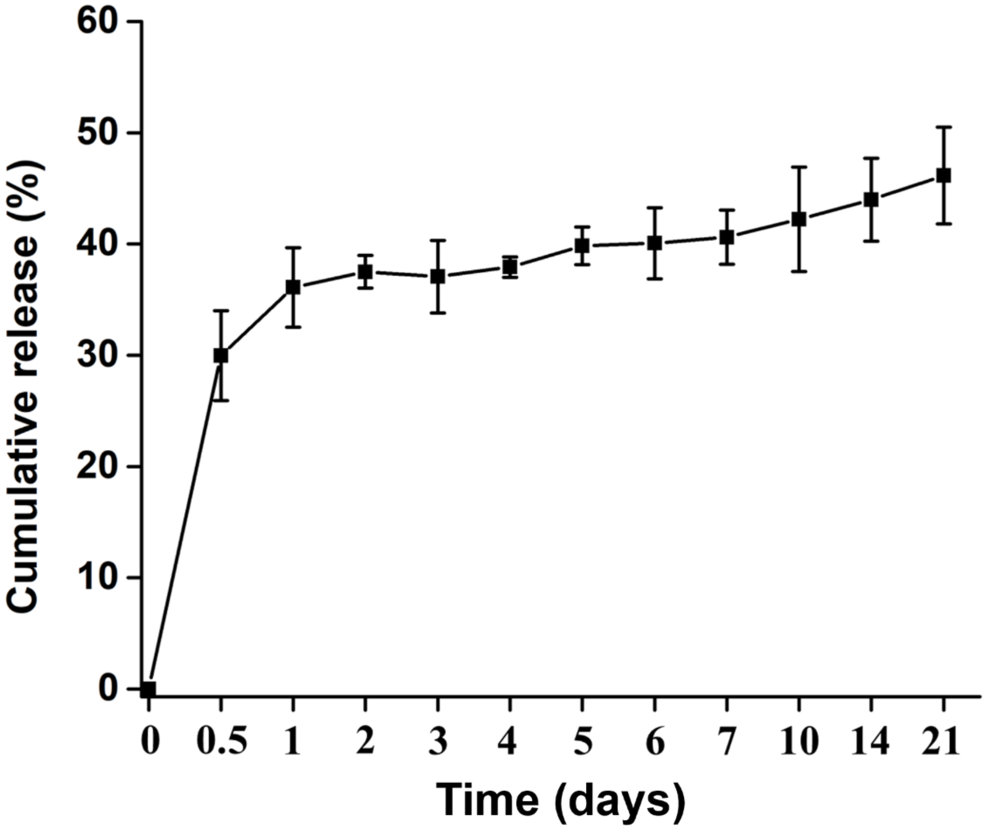


**Figure S8. *In vitro* release of adenosine.** Cumulative percentage of *in vitro* release of adenosine from PEGDA-6ACA-PBA macroporous hydrogel over 21 days (mean ± SEM, *N*=3).

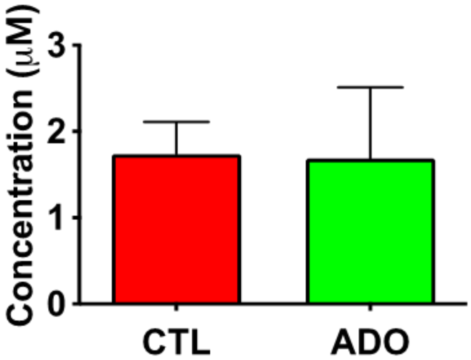


**Figure S9. Adenosine levels in circulation following implantation of adenosine-loaded macroporous hydrogels at the tibial fracture site.** Concentration of adenosine in peripheral blood of fractured mice treated with control (CTL) or adenosine (ADO)-loaded macroporous hydrogel at 3 days post-fracture (mean ± SEM, *N*=3 mice. Mann Whitney U test).

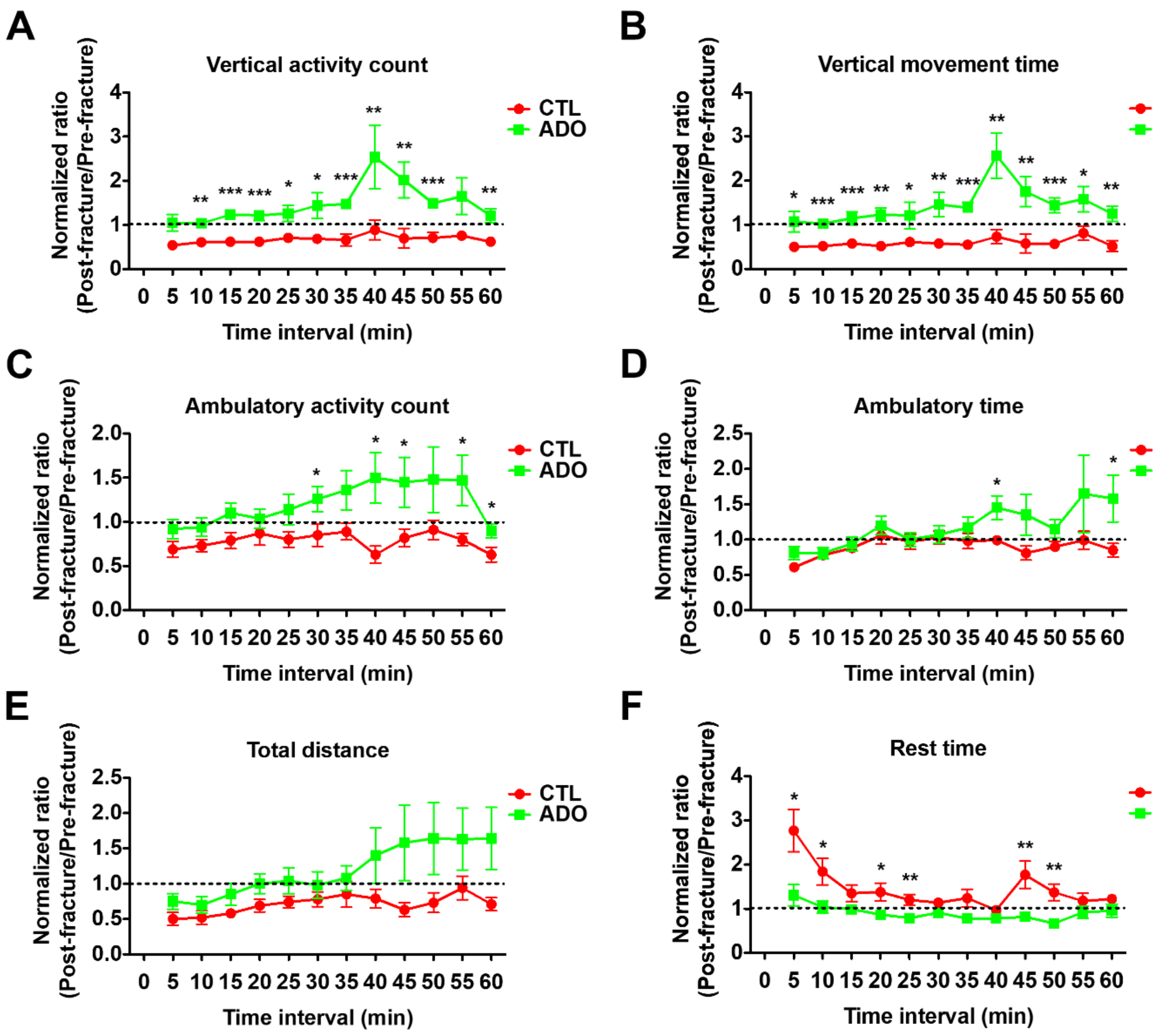


**Figure S10. Local delivery of adenosine improves open field activity of fractured animals.** Normalized ratio of (**A**) vertical activity count, (**B**) vertical movement time, (**C**) ambulatory activity count, (**D**) ambulatory time, (**E**) total distance, and (**F**) rest time of mice implanted with control (CTL) or adenosine (ADO)-loaded macroporous hydrogel at 5-min intervals at 7 days post fracture. Results of treatment are normalized to the baseline (pre-fracture) values (mean ± SEM, $N$=9 mice per group. Mann Whitney U test). *$P$<0.05, **$P$ < 0.01, ***$P$ < 0.001.

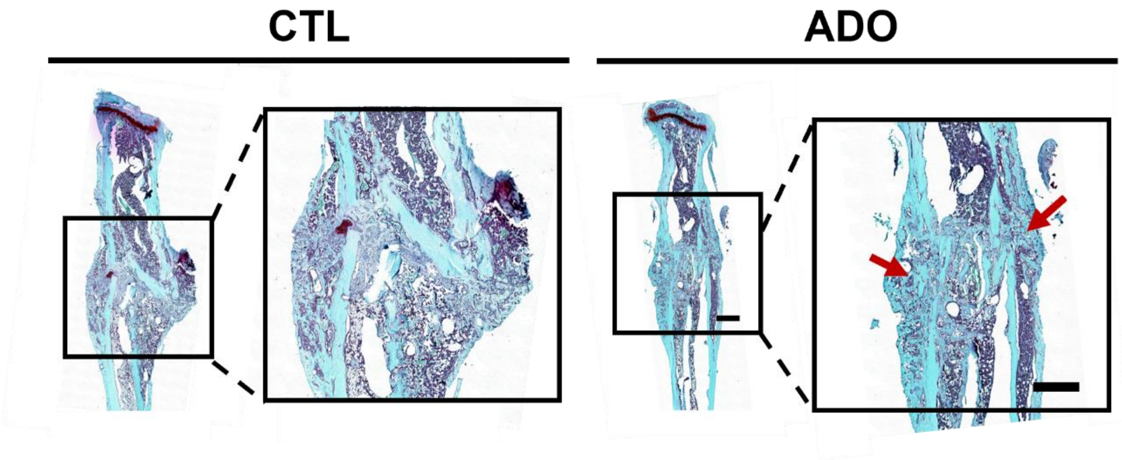


**Figure S11. Safranin O staining.** Representative Safranin O staining of fractured tibiae treated with adenosine (ADO) or control (CTL)-loaded hydrogels at 21 dpf. Red arrow indicates connected cortical bone. Scale bar, 2 mm.

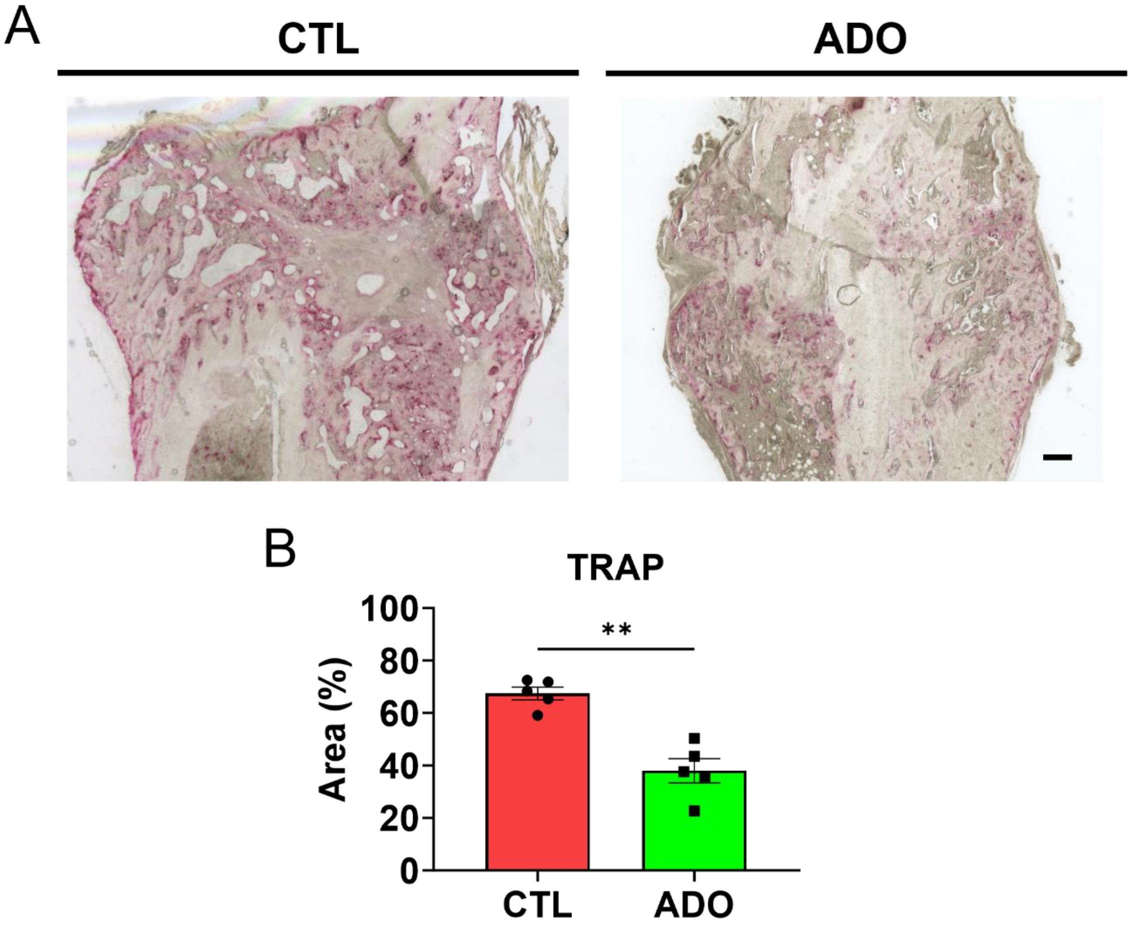


**Figure S12. TRAP staining.** (**A**) Representative TRAP staining of fractured tibiae treated with adenosine (ADO) or control (CTL)-loaded hydrogels at 21 dpf. Scale bar, 500 µm. (**B**) Quantification of TRAP stain as percent area of positive stain over total bone area (bone area, %). (mean ± SEM, *N*=5 mice per group. Mann Whitney U test). $**P < 0.01$.

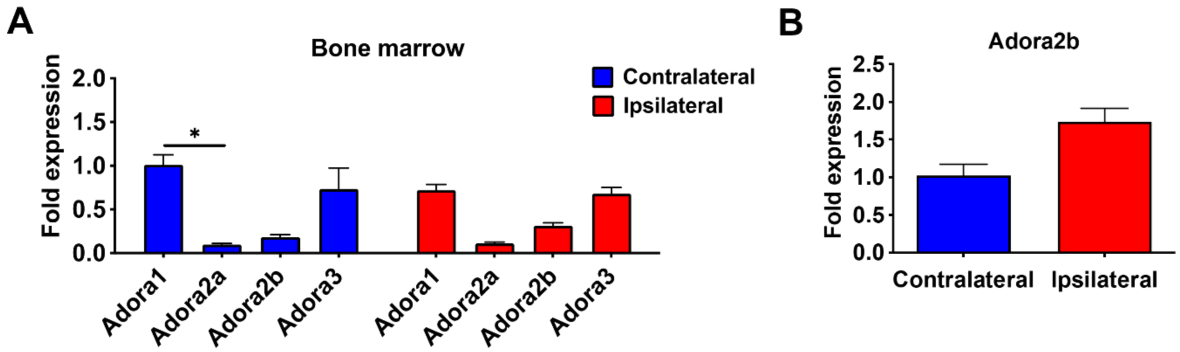


**Figure S13. Adenosine receptor gene expression of the whole bone marrow.** (**A**) Relative gene expression of adenosine receptors in whole bone marrow of fractured mice at 5 days post-fracture (mean ± SEM, *N*=3 mice per group. Kruskal-Wallis with Dunn's post hoc test was used for statistical analysis). (**B**) Relative gene expression of *Adora2b* of the bone marrow (BM) of contralateral and ipsilateral limbs (mean ± SEM, *N*=3 mice per group. Mann Whitney U test). *$P<0.05$,

## Vertical activity count

| Pre-fracture | Time interval (min) | | | | | | | | | | |
|---|---|---|---|---|---|---|---|---|---|---|---|
| **Animal** | **0-5** | **5-10** | **10-15** | **15-20** | **20-25** | **25-30** | **30-35** | **35-40** | **40-45** | **45-50** | **50-55** | **55-60** |
| CTL-1 | 100 | 125 | 120 | 122 | 102 | 171 | 144 | 131 | 110 | 140 | 82 | 98 |
| CTL-2 | 126 | 99 | 117 | 90 | 91 | 125 | 99 | 140 | 176 | 137 | 194 | 146 |
| CTL-3 | 131 | 125 | 82 | 103 | 111 | 149 | 146 | 81 | 97 | 160 | 111 | 122 |
| CTL-4 | 114 | 130 | 97 | 78 | 106 | 122 | 146 | 144 | 133 | 156 | 146 | 138 |
| CTL-5 | 84 | 82 | 99 | 114 | 106 | 47 | 89 | 111 | 64 | 107 | 61 | 78 |
| CTL-6 | 72 | 63 | 78 | 97 | 138 | 146 | 133 | 189 | 132 | 147 | 100 | 147 |
| CTL-7 | 168 | 178 | 202 | 217 | 205 | 185 | 204 | 176 | 227 | 148 | 179 | 185 |
| CTL-8 | 129 | 137 | 133 | 136 | 129 | 123 | 135 | 174 | 166 | 134 | 113 | 129 |
| CTL-9 | 71 | 107 | 96 | 118 | 109 | 98 | 98 | 40 | 51 | 103 | 136 | 141 |

| Post-fracture | Time interval (min) | | | | | | | | | | | |
|---|---|---|---|---|---|---|---|---|---|---|---|---|
| **Animal** | **0-5** | **5-10** | **10-15** | **15-20** | **20-25** | **25-30** | **30-35** | **35-40** | **40-45** | **45-50** | **50-55** | **55-60** |
| CTL-1 | 109 | 108 | 81 | 125 | 103 | 91 | 89 | 128 | 74 | 128 | 88 | 119 |
| CTL-2 | 42 | 82 | 89 | 74 | 88 | 92 | 105 | 121 | 79 | 132 | 159 | 99 |
| CTL-3 | 61 | 56 | 33 | 41 | 75 | 83 | 50 | 78 | 42 | 31 | 78 | 61 |
| CTL-4 | 44 | 43 | 51 | 36 | 32 | 44 | 30 | 41 | 12 | 33 | 28 | 20 |
| CTL-5 | 27 | 23 | 38 | 65 | 43 | 55 | 60 | 66 | 64 | 54 | 45 | 63 |
| CTL-6 | 37 | 68 | 87 | 54 | 89 | 82 | 89 | 86 | 81 | 115 | 55 | 132 |
| CTL-7 | 53 | 88 | 91 | 74 | 149 | 71 | 72 | 102 | 98 | 103 | 128 | 45 |
| CTL-8 | 70 | 65 | 74 | 87 | 67 | 146 | 71 | 122 | 44 | 116 | 144 | 60 |
| CTL-9 | 64 | 75 | 67 | 87 | 123 | 71 | 150 | 103 | 118 | 131 | 111 | 93 |

| Pre-fracture | Time interval (min) | | | | | | | | | | | |
|---|---|---|---|---|---|---|---|---|---|---|---|---|
| **Animal** | **0-5** | **5-10** | **10-15** | **15-20** | **20-25** | **25-30** | **30-35** | **35-40** | **40-45** | **45-50** | **50-55** | **55-60** |
| ADO-1 | 58 | 110 | 70 | 119 | 92 | 116 | 105 | 95 | 104 | 106 | 161 | 126 |
| ADO-2 | 77 | 64 | 36 | 47 | 58 | 31 | 68 | 56 | 30 | 62 | 91 | 87 |
| ADO-3 | 71 | 125 | 112 | 66 | 71 | 102 | 56 | 115 | 126 | 89 | 50 | 87 |
| ADO-4 | 95 | 84 | 99 | 88 | 103 | 100 | 86 | 99 | 87 | 80 | 94 | 89 |
| ADO-5 | 60 | 89 | 129 | 134 | 133 | 94 | 124 | 81 | 110 | 125 | 119 | 90 |
| ADO-6 | 106 | 67 | 73 | 75 | 113 | 94 | 58 | 22 | 22 | 63 | 12 | 55 |
| ADO-7 | 57 | 70 | 91 | 77 | 56 | 59 | 83 | 21 | 40 | 93 | 99 | 85 |
| ADO-8 | 58 | 110 | 70 | 119 | 92 | 116 | 105 | 95 | 104 | 106 | 161 | 126 |
| ADO-9 | 118 | 110 | 70 | 107 | 87 | 129 | 91 | 107 | 111 | 111 | 107 | 92 |

| Post-fracture | Time interval (min) | | | | | | | | | | | |
|---|---|---|---|---|---|---|---|---|---|---|---|---|
| **Animal** | **0-5** | **5-10** | **10-15** | **15-20** | **20-25** | **25-30** | **30-35** | **35-40** | **40-45** | **45-50** | **50-55** | **55-60** |
| ADO-1 | 114 | 107 | 125 | 122 | 134 | 84 | 126 | 154 | 132 | 107 | 117 | 111 |
| ADO-2 | 21 | 80 | 58 | 90 | 125 | 95 | 81 | 106 | 113 | 112 | 50 | 93 |
| ADO-3 | 54 | 100 | 86 | 83 | 60 | 78 | 100 | 130 | 115 | 145 | 125 | 80 |
| ADO-4 | 46 | 81 | 142 | 147 | 158 | 147 | 178 | 179 | 153 | 141 | 137 | 109 |
| ADO-5 | 99 | 121 | 107 | 107 | 107 | 154 | 168 | 184 | 161 | 170 | 132 | 166 |
| ADO-6 | 76 | 68 | 75 | 71 | 53 | 67 | 70 | 87 | 57 | 87 | 54 | 32 |
| ADO-7 | 67 | 77 | 94 | 82 | 102 | 158 | 143 | 163 | 167 | 180 | 208 | 171 |
| ADO-8 | 81 | 110 | 84 | 129 | 122 | 119 | 161 | 130 | 126 | 148 | 126 | 118 |
| ADO-9 | 126 | 97 | 96 | 135 | 81 | 118 | 105 | 114 | 119 | 122 | 120 | 131 |

**Table S1. Vertical activity count in open field locomotion.** Vertical activity count during each 5 min interval for 60 min, before and after fracture with or without adenosine treatment.

## Vertical movement time (s)

| Pre-fracture | Time interval (min) | | | | | | | | | | |
|---|---|---|---|---|---|---|---|---|---|---|---|
| **Animal** | **0-5** | **5-10** | **10-15** | **15-20** | **20-25** | **25-30** | **30-35** | **35-40** | **40-45** | **45-50** | **50-55** | **55-60** |
| CTL-1 | 35 | 35.3 | 38.9 | 39.6 | 27.7 | 55.1 | 38.5 | 40.7 | 28.9 | 43.2 | 17.2 | 22.8 |
| CTL-2 | 43.9 | 41.3 | 43.5 | 31.9 | 34.6 | 47 | 32.6 | 47.5 | 56 | 55.2 | 63.1 | 54.8 |
| CTL-3 | 45 | 48.5 | 24.8 | 32.4 | 35.7 | 45.1 | 50.8 | 29.1 | 31.9 | 52.5 | 33 | 30.7 |
| CTL-4 | 32.8 | 45.8 | 30.5 | 28.3 | 33.9 | 43.1 | 53.5 | 47.7 | 50.1 | 63.5 | 49.6 | 45.3 |
| CTL-5 | 32.2 | 31.5 | 37.7 | 40.8 | 44.9 | 19.8 | 36.7 | 44.5 | 27.9 | 48.8 | 16.5 | 29.7 |
| CTL-6 | 25.2 | 21.2 | 26.8 | 34.7 | 43.3 | 47.1 | 42 | 67.2 | 48 | 53.4 | 38.3 | 53.1 |
| CTL-7 | 48.9 | 57 | 65.1 | 72.4 | 66.3 | 63.4 | 72.9 | 69.3 | 80.2 | 53.1 | 81.4 | 66.8 |
| CTL-8 | 50.3 | 61.8 | 47.7 | 49.4 | 47.3 | 42.5 | 55.8 | 56.8 | 55.7 | 42.8 | 31.2 | 45.6 |
| CTL-9 | 21.8 | 30 | 34.5 | 37.8 | 31.3 | 29.9 | 28.4 | 13.5 | 14.7 | 33.5 | 42.5 | 40.4 |

| Post-fracture | Time interval (min) | | | | | | | | | | | |
|---|---|---|---|---|---|---|---|---|---|---|---|---|
| **Animal** | **0-5** | **5-10** | **10-15** | **15-20** | **20-25** | **25-30** | **30-35** | **35-40** | **40-45** | **45-50** | **50-55** | **55-60** |
| CTL-1 | 40.3 | 33.2 | 26.4 | 42.7 | 28 | 30.6 | 28.2 | 37 | 23.9 | 43.1 | 29.1 | 29.9 |
| CTL-2 | 9.1 | 18.7 | 22.6 | 23.1 | 26.3 | 22.5 | 25.1 | 26 | 14.4 | 27 | 40.6 | 21 |
| CTL-3 | 24 | 23.2 | 16.8 | 14.1 | 21.2 | 24.1 | 11.4 | 23.2 | 10.5 | 7.2 | 21.3 | 12.1 |
| CTL-4 | 15 | 11.7 | 14.4 | 9.2 | 11.4 | 14.5 | 12.5 | 16.1 | 4.6 | 13.3 | 10.2 | 4.4 |
| CTL-5 | 8.4 | 10.7 | 19.1 | 21.1 | 15 | 18 | 19.4 | 24.3 | 14.7 | 17 | 16 | 22.6 |
| CTL-6 | 10 | 18.9 | 21.4 | 10.3 | 24.8 | 23.7 | 27.3 | 25.3 | 24.4 | 37.9 | 16.1 | 34.9 |
| CTL-7 | 13.9 | 24.6 | 31.2 | 19.4 | 37.1 | 22.8 | 20.3 | 29.3 | 25.6 | 22.3 | 35.7 | 7.9 |
| CTL-8 | 18.8 | 19.2 | 28.1 | 25.5 | 16.7 | 45.1 | 15.4 | 38.2 | 12.1 | 33.3 | 45 | 14.3 |
| CTL-9 | 17.5 | 18.4 | 18.2 | 21 | 31.6 | 14.5 | 36.2 | 26 | 32 | 34.7 | 34.8 | 25.8 |

| Pre-fracture | Time interval (min) | | | | | | | | | | | |
|---|---|---|---|---|---|---|---|---|---|---|---|---|
| **Animal** | **0-5** | **5-10** | **10-15** | **15-20** | **20-25** | **25-30** | **30-35** | **35-40** | **40-45** | **45-50** | **50-55** | **55-60** |
| ADO-1 | 13.2 | 23 | 23.7 | 20.6 | 12.5 | 15.2 | 28.8 | 14.7 | 18.9 | 34.4 | 27.4 | 30.9 |
| ADO-2 | 23.3 | 24.5 | 16.5 | 19.6 | 27.1 | 11.2 | 17.1 | 12.8 | 10 | 14.7 | 22.3 | 26.1 |
| ADO-3 | 25.6 | 41.3 | 43.3 | 18.2 | 25.2 | 36.2 | 19.9 | 30.6 | 43.9 | 24.1 | 14.4 | 27.6 |
| ADO-4 | 33 | 27.2 | 30.5 | 25.8 | 29.3 | 30.5 | 22.9 | 31.1 | 31.9 | 23.7 | 28.3 | 19.9 |
| ADO-5 | 21.8 | 32.1 | 45.1 | 35.4 | 49.8 | 27.3 | 40.1 | 21 | 36.8 | 36.5 | 34.1 | 23.5 |
| ADO-6 | 30.6 | 19.4 | 21.5 | 26.6 | 40.7 | 29.8 | 22.4 | 5.9 | 8.1 | 23 | 5.3 | 20.9 |
| ADO-7 | 14.8 | 22.7 | 31.7 | 22.2 | 19.4 | 23.3 | 26.5 | 8 | 11.2 | 40.6 | 27.5 | 20.9 |
| ADO-8 | 22.6 | 40 | 26.7 | 43.1 | 38.9 | 48.5 | 39.6 | 38 | 42.5 | 44 | 60.1 | 37.1 |
| ADO-9 | 40.7 | 38.4 | 24.5 | 39.9 | 32.1 | 37.3 | 32.7 | 36.9 | 36.7 | 34 | 43.6 | 27.9 |

| Post-fracture | Time interval (min) | | | | | | | | | | | |
|---|---|---|---|---|---|---|---|---|---|---|---|---|
| **Animal** | **0-5** | **5-10** | **10-15** | **15-20** | **20-25** | **25-30** | **30-35** | **35-40** | **40-45** | **45-50** | **50-55** | **55-60** |
| ADO-1 | 35.3 | 37.5 | 45.5 | 36.1 | 43.3 | 28.3 | 44.6 | 46.9 | 37 | 27.7 | 44.7 | 32.7 |
| ADO-2 | 6.7 | 28.1 | 21.3 | 32.8 | 35.3 | 36.9 | 27.5 | 29.6 | 33.4 | 32.9 | 15.5 | 33.3 |
| ADO-3 | 14.9 | 29.3 | 22 | 22.6 | 14.4 | 21.3 | 26.2 | 34.3 | 32.2 | 34.4 | 34.8 | 17.8 |
| ADO-4 | 15.8 | 23.4 | 49.2 | 49.3 | 44.5 | 45 | 44.8 | 57.8 | 47.3 | 53.3 | 47.7 | 32.1 |
| ADO-5 | 26.2 | 35.4 | 36.8 | 32.9 | 32.4 | 39.3 | 56 | 60 | 42.3 | 58.5 | 36 | 43 |
| ADO-6 | 25.4 | 22.3 | 25.3 | 18.5 | 16.1 | 19.9 | 15.6 | 25.8 | 12.8 | 25.8 | 17.1 | 9.7 |
| ADO-7 | 21.1 | 21.2 | 24.1 | 20.3 | 21.9 | 41.4 | 40.4 | 42.8 | 39.4 | 52.1 | 54.5 | 42.9 |
| ADO-8 | 28.1 | 37.9 | 24.2 | 40 | 33.6 | 43.6 | 53.9 | 40 | 39.5 | 52.2 | 40.7 | 39.4 |
| ADO-9 | 37.5 | 31.2 | 33.3 | 39.1 | 30.9 | 43.1 | 34.6 | 38.3 | 39.8 | 37.1 | 38.7 | 34.8 |

**Table S2. Vertical movement time (s) in open field locomotion.** Vertical movement time (s) during each 5 min interval for 60 min, before and after fracture with or without adenosine treatment.

# Ambulatory activity count

| Pre-fracture | Time interval (min) | | | | | | | | | | | |
|---|---|---|---|---|---|---|---|---|---|---|---|---|
| **Animal** | **0-5** | **5-10** | **10-15** | **15-20** | **20-25** | **25-30** | **30-35** | **35-40** | **40-45** | **45-50** | **50-55** | **55-60** |
| CTL-1 | 1741 | 1522 | 1715 | 1244 | 991 | 1612 | 1043 | 1245 | 1444 | 1503 | 950 | 1373 |
| CTL-2 | 1652 | 1542 | 1474 | 1092 | 1195 | 1458 | 968 | 1158 | 1694 | 1586 | 1503 | 1419 |
| CTL-3 | 2018 | 1674 | 1378 | 1152 | 1425 | 1477 | 1776 | 1429 | 1328 | 1547 | 1595 | 1202 |
| CTL-4 | 1886 | 1563 | 1073 | 1005 | 1128 | 1189 | 1084 | 1488 | 1398 | 1684 | 1311 | 1023 |
| CTL-5 | 1280 | 1184 | 1218 | 1115 | 1388 | 737 | 922 | 1100 | 919 | 920 | 525 | 990 |
| CTL-6 | 1760 | 1175 | 1419 | 1205 | 1163 | 1131 | 1007 | 1169 | 1093 | 1232 | 957 | 955 |
| CTL-7 | 2082 | 1891 | 1733 | 1682 | 1375 | 1391 | 1113 | 1208 | 1224 | 1247 | 1383 | 993 |
| CTL-8 | 1834 | 1969 | 1780 | 1904 | 1686 | 1448 | 1795 | 1500 | 1660 | 1665 | 1190 | 1582 |
| CTL-9 | 1078 | 973 | 790 | 873 | 646 | 884 | 709 | 564 | 871 | 729 | 868 | 863 |

| Post-fracture | Time interval (min) | | | | | | | | | | | |
|---|---|---|---|---|---|---|---|---|---|---|---|---|
| **Animal** | **0-5** | **5-10** | **10-15** | **15-20** | **20-25** | **25-30** | **30-35** | **35-40** | **40-45** | **45-50** | **50-55** | **55-60** |
| CTL-1 | 2000 | 1804 | 1628 | 1436 | 1297 | 1130 | 1402 | 1651 | 809 | 1700 | 1378 | 1198 |
| CTL-2 | 1036 | 1422 | 1225 | 1344 | 1473 | 1194 | 981 | 1092 | 1005 | 1232 | 1594 | 1138 |
| CTL-3 | 1194 | 1093 | 958 | 774 | 886 | 973 | 771 | 926 | 905 | 809 | 955 | 900 |
| CTL-4 | 862 | 644 | 703 | 587 | 598 | 590 | 703 | 1112 | 308 | 732 | 552 | 518 |
| CTL-5 | 655 | 527 | 843 | 843 | 746 | 985 | 769 | 787 | 707 | 669 | 561 | 798 |
| CTL-6 | 794 | 787 | 839 | 826 | 916 | 795 | 951 | 1249 | 862 | 1311 | 773 | 1119 |
| CTL-7 | 784 | 792 | 824 | 739 | 880 | 777 | 605 | 656 | 714 | 733 | 756 | 676 |
| CTL-8 | 1096 | 1220 | 1028 | 1182 | 925 | 1302 | 715 | 1085 | 458 | 1320 | 1287 | 953 |
| CTL-9 | 1013 | 886 | 875 | 873 | 1026 | 870 | 1030 | 724 | 1035 | 973 | 971 | 902 |

| Pre-fracture | Time interval (min) | | | | | | | | | | | |
|---|---|---|---|---|---|---|---|---|---|---|---|---|
| **Animal** | **0-5** | **5-10** | **10-15** | **15-20** | **20-25** | **25-30** | **30-35** | **35-40** | **40-45** | **45-50** | **50-55** | **55-60** |
| ADO-1 | 1200 | 1423 | 1267 | 1265 | 785 | 807 | 870 | 978 | 1045 | 1280 | 1206 | 1292 |
| ADO-2 | 1153 | 1114 | 817 | 832 | 953 | 551 | 960 | 763 | 327 | 609 | 918 | 425 |
| ADO-3 | 1077 | 1149 | 1031 | 659 | 681 | 890 | 697 | 768 | 895 | 491 | 250 | 392 |
| ADO-4 | 1224 | 1041 | 1047 | 742 | 996 | 668 | 697 | 582 | 716 | 378 | 623 | 486 |
| ADO-5 | 1173 | 1050 | 1159 | 1088 | 1056 | 802 | 823 | 513 | 870 | 848 | 996 | 1010 |
| ADO-6 | 1664 | 1325 | 1223 | 1273 | 1307 | 1516 | 1426 | 1159 | 872 | 1225 | 766 | 1069 |
| ADO-7 | 1078 | 1182 | 1484 | 945 | 986 | 957 | 985 | 845 | 494 | 856 | 1190 | 698 |
| ADO-8 | 1398 | 2024 | 1541 | 1599 | 1434 | 1591 | 1112 | 1327 | 1494 | 1631 | 1502 | 1567 |
| ADO-9 | 1983 | 1518 | 1088 | 1234 | 1014 | 1029 | 877 | 826 | 1134 | 945 | 649 | 532 |

| Post-fracture | Time interval (min) | | | | | | | | | | | |
|---|---|---|---|---|---|---|---|---|---|---|---|---|
| **Animal** | **0-5** | **5-10** | **10-15** | **15-20** | **20-25** | **25-30** | **30-35** | **35-40** | **40-45** | **45-50** | **50-55** | **55-60** |
| ADO-1 | 1488 | 1204 | 1124 | 1208 | 1123 | 1044 | 1070 | 1072 | 1171 | 928 | 1032 | 776 |
| ADO-2 | 839 | 1417 | 1250 | 1169 | 1344 | 1127 | 1037 | 1150 | 1023 | 968 | 708 | 1384 |
| ADO-3 | 956 | 1080 | 810 | 831 | 720 | 712 | 976 | 885 | 899 | 1009 | 1072 | 456 |
| ADO-4 | 956 | 1039 | 1165 | 1137 | 1019 | 1064 | 1081 | 1039 | 985 | 1209 | 978 | 850 |
| ADO-5 | 1382 | 1610 | 1456 | 1506 | 1396 | 1189 | 1531 | 1460 | 1327 | 1668 | 1449 | 1445 |
| ADO-6 | 1093 | 685 | 735 | 648 | 603 | 606 | 596 | 565 | 643 | 633 | 575 | 448 |
| ADO-7 | 1105 | 1021 | 891 | 1113 | 840 | 1156 | 1172 | 1074 | 1359 | 896 | 1247 | 1270 |
| ADO-8 | 1492 | 1483 | 1294 | 1472 | 1546 | 1218 | 1806 | 1468 | 1491 | 1549 | 1355 | 1357 |
| ADO-9 | 1151 | 820 | 906 | 926 | 760 | 701 | 847 | 822 | 959 | 983 | 1106 | 1021 |

**Table S3. Ambulatory activity count in open field locomotion.** Ambulatory activity during each 5 min interval for 60 min, before and after fracture with or without adenosine treatment.

## Ambulatory time (s)

| Pre-fracture | Time interval (min) | | | | | | | | | | |
|---|---|---|---|---|---|---|---|---|---|---|---|
| **Animal** | **0-5** | **5-10** | **10-15** | **15-20** | **20-25** | **25-30** | **30-35** | **35-40** | **40-45** | **45-50** | **50-55** | **55-60** |
| CTL-1 | 17.6 | 15.2 | 16.1 | 10.8 | 12.5 | 18 | 11.6 | 13.8 | 14.9 | 13.6 | 14.7 | 15.4 |
| CTL-2 | 21.3 | 16.2 | 16.7 | 10.1 | 11.4 | 17.5 | 11.4 | 12.7 | 19.9 | 16.4 | 17.7 | 17.8 |
| CTL-3 | 25.3 | 18.8 | 13.4 | 14.5 | 18.8 | 15.3 | 18.5 | 13.8 | 12.8 | 18.4 | 17.4 | 12 |
| CTL-4 | 25.4 | 14.5 | 10.9 | 6.7 | 13.5 | 10.4 | 15.1 | 15.1 | 11.6 | 17 | 14.3 | 9.8 |
| CTL-5 | 16.3 | 8.7 | 14.6 | 10.7 | 13.3 | 8.1 | 10.4 | 13.4 | 12 | 11.8 | 5.8 | 12.8 |
| CTL-6 | 22.4 | 10.5 | 18.1 | 15.3 | 13.9 | 11.8 | 12 | 15.6 | 11.1 | 16.8 | 14.4 | 16.2 |
| CTL-7 | 26.5 | 18.7 | 16.3 | 20.5 | 15.9 | 15.2 | 12.2 | 10.8 | 15.3 | 15.8 | 15 | 12.2 |
| CTL-8 | 31.5 | 26.5 | 19.7 | 21.6 | 20.3 | 15.5 | 15.7 | 17.2 | 18 | 18.8 | 14 | 16.2 |
| CTL-9 | 20 | 16.5 | 13 | 14.6 | 10.6 | 14 | 12.7 | 9.6 | 13.8 | 11.9 | 15.6 | 16.1 |

| Post-fracture | Time interval (min) | | | | | | | | | | | |
|---|---|---|---|---|---|---|---|---|---|---|---|---|
| **Animal** | **0-5** | **5-10** | **10-15** | **15-20** | **20-25** | **25-30** | **30-35** | **35-40** | **40-45** | **45-50** | **50-55** | **55-60** |
| CTL-1 | 17.6 | 13.5 | 16.3 | 11.9 | 13.3 | 12.3 | 16 | 16.7 | 11.4 | 17.5 | 13.2 | 7.9 |
| CTL-2 | 13.9 | 13.8 | 9.8 | 13.7 | 15.2 | 16.1 | 10.4 | 12.6 | 11.6 | 14.2 | 16.4 | 12.2 |
| CTL-3 | 14.7 | 12.8 | 13.4 | 12.6 | 15.5 | 15.2 | 11.8 | 12.3 | 12.7 | 11.3 | 15.8 | 17.9 |
| CTL-4 | 10.7 | 8.1 | 9.7 | 11.6 | 10.3 | 8.1 | 9.8 | 15.4 | 4 | 11.9 | 7.9 | 7.9 |
| CTL-5 | 11.1 | 9 | 15.5 | 14.5 | 15.3 | 13 | 16 | 12 | 11.1 | 11.3 | 10.4 | 12.5 |
| CTL-6 | 10.7 | 8.3 | 12.1 | 9.7 | 9.7 | 10.9 | 13 | 13.4 | 12.4 | 14.4 | 13.4 | 12.5 |
| CTL-7 | 13.3 | 13.5 | 14 | 15 | 15.7 | 15.1 | 7.5 | 10.1 | 9.7 | 11.5 | 8.1 | 7.3 |
| CTL-8 | 17.6 | 12.7 | 16.5 | 15.7 | 9.7 | 19 | 13.8 | 14.1 | 11.5 | 16.2 | 14.5 | 14.6 |
| CTL-9 | 13.1 | 16.5 | 12.8 | 14.9 | 15.3 | 16 | 13.8 | 12.5 | 18.2 | 14.4 | 20.2 | 14.5 |

| Pre-fracture | Time interval (min) | | | | | | | | | | | |
|---|---|---|---|---|---|---|---|---|---|---|---|---|
| **Animal** | **0-5** | **5-10** | **10-15** | **15-20** | **20-25** | **25-30** | **30-35** | **35-40** | **40-45** | **45-50** | **50-55** | **55-60** |
| ADO-1 | 13.2 | 15.4 | 13.6 | 9.8 | 10.1 | 9.2 | 10.8 | 9.8 | 12.7 | 11.3 | 10.7 | 10 |
| ADO-2 | 19.3 | 12.1 | 10.2 | 11.6 | 10.5 | 8.1 | 15 | 10.3 | 3.7 | 7.9 | 15.9 | 3.9 |
| ADO-3 | 17.3 | 12.6 | 16.1 | 8.2 | 9.7 | 14.9 | 5.1 | 10.2 | 15 | 10.8 | 2.4 | 5.2 |
| ADO-4 | 18.7 | 14.7 | 15.6 | 9.4 | 17 | 8.7 | 9.3 | 5.8 | 9.9 | 5.9 | 7.4 | 8.6 |
| ADO-5 | 13.6 | 15.4 | 17.2 | 9.2 | 14.5 | 9.5 | 10.1 | 5.1 | 11.2 | 12.2 | 13.4 | 14.7 |
| ADO-6 | 31.6 | 18.6 | 21 | 24.6 | 23.4 | 25.7 | 28.4 | 15.1 | 9.8 | 20.8 | 10.5 | 12.4 |
| ADO-7 | 14.8 | 15.5 | 19.2 | 11.5 | 12.4 | 12.5 | 11.3 | 12.6 | 6.6 | 11.3 | 13.5 | 7.3 |
| ADO-8 | 19 | 23.5 | 17.5 | 16 | 16.6 | 16.5 | 11.8 | 13.1 | 17.8 | 18.6 | 16.5 | 15 |
| ADO-9 | 24.9 | 16.8 | 9.9 | 18.4 | 12.1 | 10.4 | 10.9 | 11.4 | 17.3 | 10.7 | 9 | 6.7 |

| Post-fracture | Time interval (min) | | | | | | | | | | | |
|---|---|---|---|---|---|---|---|---|---|---|---|---|
| **Animal** | **0-5** | **5-10** | **10-15** | **15-20** | **20-25** | **25-30** | **30-35** | **35-40** | **40-45** | **45-50** | **50-55** | **55-60** |
| ADO-1 | 16.4 | 17.1 | 15.4 | 10.4 | 14.1 | 10.6 | 13 | 14.9 | 11.8 | 12 | 11.6 | 10.7 |
| ADO-2 | 13.6 | 11.2 | 13.3 | 14.2 | 12.2 | 13.3 | 14.5 | 15.7 | 11.4 | 8.5 | 8.4 | 14.4 |
| ADO-3 | 11.7 | 12.8 | 13.1 | 11.6 | 8.5 | 8.4 | 8.9 | 12.1 | 11.8 | 11.5 | 14.3 | 6.7 |
| ADO-4 | 13.5 | 7.6 | 13.3 | 17.5 | 11.2 | 10.7 | 6.8 | 11.9 | 7.5 | 11.1 | 9.9 | 10.3 |
| ADO-5 | 15.1 | 15.7 | 11.2 | 14.1 | 17.8 | 11.6 | 15.7 | 12.4 | 11.2 | 17.5 | 17.4 | 11.7 |
| ADO-6 | 13.7 | 11.1 | 12.9 | 11.4 | 10.8 | 13.1 | 10.9 | 12.8 | 14.2 | 11.1 | 12.5 | 8.7 |
| ADO-7 | 17 | 13.8 | 15.3 | 14 | 14.8 | 13.5 | 13.7 | 14.9 | 16.8 | 12.1 | 15.3 | 14.8 |
| ADO-8 | 14.2 | 15.5 | 14.2 | 17.1 | 15.8 | 13.6 | 19.6 | 17.5 | 16.4 | 15 | 14.5 | 14.3 |
| ADO-9 | 13.3 | 9 | 14.3 | 17.1 | 13.5 | 14.6 | 11.4 | 10.9 | 12.2 | 15.4 | 12.6 | 16.7 |

**Table S4. Total ambulatory time (s) in open field locomotion.** Total ambulatory time (s) during each 5 min interval for 60 min, before and after fracture with or without adenosine treatment.

# Total distance (cm)

| **Pre-fracture** | **Time interval (min)** | | | | | | | | | | | |
|---|---|---|---|---|---|---|---|---|---|---|---|---|
| **Animal** | **0-5** | **5-10** | **10-15** | **15-20** | **20-25** | **25-30** | **30-35** | **35-40** | **40-45** | **45-50** | **50-55** | **55-60** |
| CTL-1 | 438.7 | 251.8 | 281 | 210.5 | 167.9 | 271.4 | 163.5 | 167.8 | 197.3 | 178.1 | 122.4 | 239.6 |
| CTL-2 | 503.4 | 300.9 | 319.4 | 197.2 | 209.6 | 267.4 | 158 | 220.2 | 299.3 | 240.7 | 280.8 | 335.9 |
| CTL-3 | 670.1 | 413.2 | 247.8 | 220.7 | 316.4 | 329.9 | 345.5 | 230.9 | 185.3 | 300.4 | 222.6 | 199.3 |
| CTL-4 | 637.2 | 299.2 | 154.5 | 146.7 | 172.8 | 128.5 | 151.3 | 193.6 | 130.3 | 195 | 115.1 | 110.3 |
| CTL-5 | 272 | 202 | 189 | 193.6 | 243.2 | 111.6 | 143 | 193.3 | 136.5 | 152.3 | 78.3 | 212.5 |
| CTL-6 | 634.2 | 222.9 | 371.5 | 290.5 | 275.5 | 277 | 255.4 | 320.7 | 260.2 | 311.5 | 227.5 | 249.8 |
| CTL-7 | 638.2 | 410.1 | 336.2 | 336 | 236.3 | 234.5 | 206.7 | 218.8 | 304.1 | 272.2 | 306.3 | 206.8 |
| CTL-8 | 868.8 | 616.7 | 389.8 | 429.7 | 377.2 | 269.3 | 354.4 | 289.1 | 265.5 | 338.7 | 154.8 | 198.3 |
| CTL-9 | 364 | 237 | 170.5 | 203.7 | 148.3 | 191.5 | 123.2 | 119.2 | 212.6 | 177.5 | 239.7 | 260 |

| **Post-fracture** | **Time interval (min)** | | | | | | | | | | | |
|---|---|---|---|---|---|---|---|---|---|---|---|---|
| **Animal** | **0-5** | **5-10** | **10-15** | **15-20** | **20-25** | **25-30** | **30-35** | **35-40** | **40-45** | **45-50** | **50-55** | **55-60** |
| CTL-1 | 485 | 283.1 | 213.9 | 201.2 | 182 | 138.5 | 272.7 | 301.9 | 135 | 314.7 | 247.1 | 188.9 |
| CTL-2 | 285.5 | 216.8 | 162.3 | 167.7 | 197.3 | 200.6 | 150.6 | 161.5 | 158.4 | 174.4 | 317.9 | 115.4 |
| CTL-3 | 231.9 | 140.7 | 142.9 | 128.4 | 166.5 | 176.4 | 121.8 | 150.6 | 142.2 | 120.5 | 198.7 | 190.9 |
| CTL-4 | 191.6 | 61.5 | 68.8 | 81.2 | 97.3 | 86.3 | 93.8 | 157.6 | 33.6 | 91.9 | 59.5 | 56.6 |
| CTL-5 | 165.1 | 62.1 | 156.3 | 150.9 | 165.5 | 140.7 | 138.7 | 114.8 | 140.4 | 107.2 | 98 | 127.2 |
| CTL-6 | 204.1 | 124.9 | 154 | 123.9 | 144.7 | 127.7 | 123.9 | 191.8 | 157.7 | 203.8 | 119.6 | 186.8 |
| CTL-7 | 177.9 | 141.3 | 119.5 | 145.4 | 165.8 | 146.8 | 101.9 | 102 | 149.5 | 101.9 | 119.2 | 113.5 |
| CTL-8 | 285.7 | 239.2 | 176.1 | 194.4 | 192.9 | 261.4 | 101.4 | 156.4 | 59.4 | 210.3 | 164.1 | 254.2 |
| CTL-9 | 242.6 | 166.6 | 152.7 | 236.1 | 168.3 | 244.6 | 220.6 | 108.1 | 227.7 | 154.9 | 171.5 | 170 |

| **Pre-fracture** | **Time interval (min)** | | | | | | | | | | | |
|---|---|---|---|---|---|---|---|---|---|---|---|---|
| **Animal** | **0-5** | **5-10** | **10-15** | **15-20** | **20-25** | **25-30** | **30-35** | **35-40** | **40-45** | **45-50** | **50-55** | **55-60** |
| ADO-1 | 258.3 | 198.3 | 234.5 | 211.1 | 112.6 | 124.4 | 160.4 | 119 | 162 | 171.5 | 94.1 | 129.1 |
| ADO-2 | 404.7 | 289.8 | 96.3 | 150.4 | 146 | 88.4 | 174.3 | 99 | 26.4 | 92.2 | 264.1 | 54.4 |
| ADO-3 | 348.7 | 194.4 | 235.6 | 87.6 | 134.3 | 161.9 | 86.9 | 191.8 | 249.3 | 81.2 | 38.3 | 84.8 |
| ADO-4 | 436.9 | 227.5 | 208.3 | 132.8 | 211.3 | 111.1 | 114.5 | 68.4 | 124.8 | 43.9 | 93.1 | 85.7 |
| ADO-5 | 289.1 | 205.4 | 191.4 | 198.3 | 180.5 | 134.3 | 147.5 | 56.3 | 115.7 | 155.5 | 214.8 | 198.3 |
| ADO-6 | 666.5 | 405.1 | 418 | 428.4 | 433.2 | 503.9 | 480.7 | 280 | 236.2 | 357.1 | 182.4 | 202 |
| ADO-7 | 339.6 | 306.7 | 311.1 | 198.1 | 219.1 | 200.1 | 192 | 192.9 | 113 | 137.6 | 202 | 97.3 |
| ADO-8 | 427.8 | 531.1 | 286.3 | 429.2 | 247.4 | 302 | 232.2 | 279.9 | 323.9 | 386.7 | 273.8 | 303.8 |
| ADO-9 | 536.3 | 271 | 189.4 | 256.1 | 178.7 | 214.6 | 182.7 | 200 | 224.2 | 209.2 | 136.2 | 93.2 |

| **Post-fracture** | **Time interval (min)** | | | | | | | | | | | |
|---|---|---|---|---|---|---|---|---|---|---|---|---|
| **Animal** | **0-5** | **5-10** | **10-15** | **15-20** | **20-25** | **25-30** | **30-35** | **35-40** | **40-45** | **45-50** | **50-55** | **55-60** |
| ADO-1 | 366.1 | 268 | 186.4 | 195.8 | 213.7 | 144.1 | 154.2 | 163.5 | 193.7 | 125.6 | 162.4 | 99.3 |
| ADO-2 | 246.8 | 191.4 | 181 | 220.3 | 218.3 | 187.9 | 134.8 | 173.4 | 146.4 | 169.2 | 101.1 | 251.3 |
| ADO-3 | 224.1 | 125.8 | 133.9 | 132.7 | 112.8 | 89.2 | 148.9 | 118.8 | 162.6 | 176.8 | 184.4 | 58.8 |
| ADO-4 | 267.2 | 133 | 200 | 176.6 | 129.4 | 121.9 | 106.7 | 132.5 | 112 | 228.5 | 197.7 | 142.3 |
| ADO-5 | 337.5 | 251.4 | 236.6 | 241.2 | 309.9 | 196.5 | 288.3 | 234.9 | 200.3 | 358.8 | 298.3 | 253 |
| ADO-6 | 228.2 | 111.3 | 97.9 | 114.5 | 122.5 | 112.9 | 121.4 | 81.4 | 121.4 | 98.2 | 102.1 | 78.5 |
| ADO-7 | 253.9 | 173.3 | 162.7 | 185.7 | 150.5 | 183.1 | 197.6 | 194.7 | 267.1 | 143.1 | 301.4 | 234.3 |
| ADO-8 | 263.7 | 260.3 | 208 | 287.5 | 230.8 | 219 | 322.3 | 245 | 209.9 | 205.5 | 220.5 | 227.7 |
| ADO-9 | 306.1 | 127.7 | 139.1 | 175.5 | 158.8 | 118.3 | 129.3 | 121 | 156.6 | 146.1 | 183.6 | 202.6 |

**Table S5. Total distance traveled (cm) in open field locomotion.** Total distance traveled (cm) during each 5 min interval for 60 min, before and after fracture with or without adenosine treatment.

## Rest time (s)

| Pre-fracture | Time interval (min) | | | | | | | | | | |
|---|---|---|---|---|---|---|---|---|---|---|---|
| **Animal** | **0-5** | **5-10** | **10-15** | **15-20** | **20-25** | **25-30** | **30-35** | **35-40** | **40-45** | **45-50** | **50-55** | **55-60** |
| CTL-1 | 45.9 | 58.2 | 66.8 | 96.4 | 104.6 | 58.4 | 91.7 | 84.3 | 80.2 | 61.7 | 99.3 | 72.1 |
| CTL-2 | 45 | 56.8 | 59 | 87.9 | 82.4 | 65 | 126.7 | 101.1 | 37 | 59.1 | 54.4 | 49.6 |
| CTL-3 | 20.8 | 36.8 | 72 | 91.1 | 64.5 | 77.2 | 46.1 | 77.6 | 80.3 | 51.1 | 43.9 | 93.7 |
| CTL-4 | 36.4 | 62.1 | 108.3 | 107.9 | 101 | 103.8 | 95.8 | 60.5 | 70 | 50.3 | 84.7 | 110.7 |
| CTL-5 | 68.9 | 85.8 | 72.1 | 103.4 | 78.7 | 115.6 | 110.9 | 94.3 | 111.7 | 96.4 | 153.5 | 88.6 |
| CTL-6 | 41.7 | 86.8 | 72.9 | 57.5 | 84.8 | 93.6 | 104.1 | 85.8 | 102.2 | 88.3 | 111.3 | 96.3 |
| CTL-7 | 21.2 | 40.9 | 37.8 | 61.2 | 67 | 77.9 | 98.9 | 112.8 | 105.9 | 81.1 | 69.7 | 104.9 |
| CTL-8 | 15.9 | 21.9 | 51.7 | 31.5 | 53.5 | 77.9 | 41.2 | 71.4 | 42.3 | 51.7 | 78.7 | 48.4 |
| CTL-9 | 59.3 | 68.3 | 95 | 90.7 | 111 | 73.8 | 113 | 128.7 | 79.3 | 119.3 | 99.2 | 93.9 |

| Post-fracture | Time interval (min) | | | | | | | | | | | |
|---|---|---|---|---|---|---|---|---|---|---|---|---|
| **Animal** | **0-5** | **5-10** | **10-15** | **15-20** | **20-25** | **25-30** | **30-35** | **35-40** | **40-45** | **45-50** | **50-55** | **55-60** |
| CTL-1 | 33.7 | 46.8 | 47.6 | 70.2 | 80.9 | 68.3 | 70.3 | 70 | 116.4 | 48.3 | 68.4 | 92.8 |
| CTL-2 | 107.3 | 74.5 | 80.6 | 93.5 | 60 | 86 | 102.3 | 87.8 | 104.5 | 102.4 | 65 | 83.5 |
| CTL-3 | 64.2 | 90.4 | 82.1 | 103.2 | 89.6 | 83 | 101.1 | 81.6 | 101.8 | 91.6 | 90.6 | 94.6 |
| CTL-4 | 115.9 | 125.6 | 130.9 | 130.8 | 149.2 | 138.7 | 129.6 | 79.7 | 185.5 | 127.3 | 143.9 | 124.8 |
| CTL-5 | 131.7 | 141 | 81.6 | 100.8 | 97.7 | 81.8 | 104.2 | 93.1 | 119.3 | 116.8 | 130.2 | 100.3 |
| CTL-6 | 116.4 | 122.6 | 116.9 | 114.9 | 107.3 | 117.3 | 106.8 | 81.2 | 115.7 | 77.9 | 112.8 | 96.5 |
| CTL-7 | 98.1 | 76.1 | 99.8 | 123.8 | 103.3 | 115.7 | 128.1 | 123 | 128.8 | 120.5 | 119.4 | 139.9 |
| CTL-8 | 80.8 | 84.4 | 80.6 | 75.8 | 88 | 62.2 | 92.8 | 61.2 | 144.8 | 58.3 | 59.4 | 77 |
| CTL-9 | 66.3 | 78.6 | 78.5 | 83.8 | 78.5 | 83.1 | 59.2 | 105.7 | 69 | 90.5 | 68.2 | 72.2 |

| Pre-fracture | Time interval (min) | | | | | | | | | | | |
|---|---|---|---|---|---|---|---|---|---|---|---|---|
| **Animal** | **0-5** | **5-10** | **10-15** | **15-20** | **20-25** | **25-30** | **30-35** | **35-40** | **40-45** | **45-50** | **50-55** | **55-60** |
| ADO-1 | 96.9 | 90.5 | 90 | 83.1 | 128.4 | 140.3 | 125.5 | 102.2 | 121.4 | 81.6 | 62 | 68.2 |
| ADO-2 | 60.8 | 92.4 | 111.5 | 127.5 | 111.5 | 151.8 | 97.4 | 125 | 169.3 | 153.5 | 84.6 | 174.5 |
| ADO-3 | 80.8 | 90.6 | 85.5 | 110.9 | 120.3 | 79.9 | 120.8 | 110 | 90.6 | 142.8 | 191.2 | 171.5 |
| ADO-4 | 81.9 | 100.2 | 94.8 | 133.5 | 114.9 | 143.2 | 145.5 | 142.2 | 127.7 | 177 | 146 | 150.9 |
| ADO-5 | 97.6 | 105.4 | 90.2 | 102.6 | 95.8 | 115 | 116.7 | 153.6 | 114.6 | 102.4 | 93.9 | 66.8 |
| ADO-6 | 93 | 121.9 | 134.6 | 126.2 | 120.6 | 101.3 | 96.7 | 96.1 | 130 | 105.4 | 141.4 | 127 |
| ADO-7 | 68.6 | 92.3 | 73.3 | 127.7 | 96.9 | 110.5 | 130.7 | 114.4 | 157.5 | 121.8 | 86.2 | 154.8 |
| ADO-8 | 58.1 | 39.6 | 73.3 | 63.3 | 71.3 | 61.1 | 96.1 | 102.5 | 64.6 | 67.5 | 53.5 | 48.3 |
| ADO-9 | 26.3 | 59.9 | 102.3 | 62.3 | 126.1 | 101.9 | 128.4 | 123 | 92 | 131.3 | 128.9 | 148.6 |

| Post-fracture | Time interval (min) | | | | | | | | | | | |
|---|---|---|---|---|---|---|---|---|---|---|---|---|
| **Animal** | **0-5** | **5-10** | **10-15** | **15-20** | **20-25** | **25-30** | **30-35** | **35-40** | **40-45** | **45-50** | **50-55** | **55-60** |
| ADO-1 | 50.1 | 68.8 | 77.1 | 61.3 | 74.8 | 86.8 | 71.8 | 86.2 | 83.9 | 58.5 | 79.8 | 110 |
| ADO-2 | 105.1 | 64.2 | 91.9 | 96.7 | 74.9 | 103.8 | 108.3 | 106.5 | 118.6 | 113.6 | 146.8 | 88.5 |
| ADO-3 | 91.4 | 86.6 | 113.6 | 106.1 | 117.8 | 128.2 | 99.4 | 76.7 | 85.5 | 99.7 | 78.1 | 162.1 |
| ADO-4 | 91.7 | 96.7 | 89.8 | 86.2 | 101.2 | 89.4 | 91.4 | 101.6 | 92.6 | 72 | 118 | 119.5 |
| ADO-5 | 75.6 | 52.5 | 53.5 | 70 | 62.6 | 79.9 | 74.7 | 54.1 | 86.8 | 37.2 | 56.8 | 68.8 |
| ADO-6 | 106.4 | 115 | 116.6 | 119 | 118.8 | 113.2 | 128.3 | 117.3 | 149.4 | 102.3 | 117.6 | 159.3 |
| ADO-7 | 85.3 | 114 | 106.5 | 97 | 90.9 | 77.8 | 95.5 | 100.7 | 70.1 | 98.2 | 71.1 | 66 |
| ADO-8 | 66.6 | 68.6 | 82.8 | 52.2 | 41.3 | 72.7 | 41.6 | 66.9 | 66.7 | 55.1 | 63.4 | 78 |
| ADO-9 | 78.4 | 94.6 | 95.6 | 97.3 | 104.2 | 99.1 | 100.3 | 103 | 84.4 | 64.3 | 72.9 | 74.5 |

**Table S6. Rest time (s) in open field locomotion.** Rest time (s) during each 5 min interval for 60 min, before and after fracture with or without adenosine treatment.
